# Valproic acid targets IDH1 mutants through alteration of lipid metabolism

**DOI:** 10.1038/s44324-024-00021-6

**Published:** 2024-08-13

**Authors:** Lubayna S. Elahi, Michael C. Condro, Riki Kawaguchi, Yue Qin, Alvaro G. Alvarado, Brandon Gruender, Haocheng Qi, Tie Li, Albert Lai, Maria G. Castro, Pedro R. Lowenstein, Matthew C. Garrett, Harley I. Kornblum

**Affiliations:** 1 Department of Psychiatry and Behavioral Sciences and the UCLA Intellectual and Developmental Disabilities Research Center, David Geffen School of Medicine, UCLA, Los Angeles, CA USA; 2 Department of Neurology, David Geffen School of Medicine, UCLA, Los Angeles, CA USA; 3 Department of Neurosurgery, Department of Cell and Developmental Biology, and Rogel Cancer Center, University of Michigan Medical School, Ann Arbor, MI USA; 4 Kettering Health Network, Kettering, OH USA; 5 Department of Molecular and Medical Pharmacology, David Geffen School of Medicine, UCLA, Los Angeles, CA USA

**Keywords:** Cancer metabolism, Oncology

## Abstract

Histone deacetylases (HDACs) have a wide range of targets and can rewire both the chromatin and lipidome of cancer cells. In this study, we show that valproic acid (VPA), a brain penetrant anti-seizure medication and histone deacetylase inhibitor, inhibits the growth of IDH1 mutant tumors in vivo and in vitro, with at least some selectivity over IDH1 wild-type tumors. Surprisingly, genes upregulated by VPA showed no enhanced chromatin accessibility at the promoter, but there was a correlation between VPA-downregulated genes and diminished promoter chromatin accessibility. VPA inhibited the transcription of lipogenic genes and these lipogenic genes showed significant decreases in promoter chromatin accessibility only in the IDH1 MT glioma cell lines tested. VPA inhibited the mTOR pathway and a key lipogenic gene, fatty acid synthase (FASN). Both VPA and a selective FASN inhibitor TVB-2640 rewired the lipidome and promoted apoptosis in an IDH1 MT but not in an IDH1 WT glioma cell line. We further find that HDACs are involved in the regulation of lipogenic genes and HDAC6 is particularly important for the regulation of FASN in IDH1 MT glioma. Finally, we show that FASN knockdown alone and VPA in combination with FASN knockdown significantly improved the survival of mice in an IDH1 MT primary orthotopic xenograft model in vivo. We conclude that targeting fatty acid metabolism through HDAC inhibition and/or FASN inhibition may be a novel therapeutic opportunity in IDH1 mutant gliomas.

## Introduction

Missense mutations in the gene isocitrate dehydrogenase one (IDH1) reprogram the metabolic and epigenetic landscape of IDH1 mutant (MT) gliomas making them molecularly and physiologically distinct from IDH1 wildtype (WT) gliomas. WT IDH1 catalyzes the conversion of isocitrate to alpha-ketoglutarate (α-kg), whereas MT IDH1 reduces α-kg to the oncometabolite 2-hydroxyglutarate (2-HG). 2-HG, by blocking α-kg, inhibits the enzymatic function of the ten-eleven translocation family of 5-methylcytosine (Tet) hydroxylases, and the Jumanji family of histone demethylases, resulting in an increase in both histone and DNA methylation^1,2^. Inhibitors that directly target MT IDH1 and block 2-HG have efficacy in low-grade IDH1 mutant gliomas but have not been proven to be beneficial for mutant high-grade gliomas^3–6^.

Chromatin-modifying drugs such as histone deacetylase inhibitors (HDACis) are promising candidates for therapeutic approaches for gliomas and have been shown to inhibit the growth of glioma by multiple mechanisms such as cell cycle arrest, apoptosis, demethylation, and metabolic rewiring^7–9^. Broad spectrum HDACi’s target histones and alter the chromatin but can also target non-histone proteins, which in an HDAC-dependent or independent manner can alter cellular signaling and metabolism^7^. In recent years, one non-histone protein that has emerged as an interesting target of HDACis is fatty acid synthase (FASN). Acetylation destabilizes the FASN protein and results in the inhibition of de novo lipogenesis^10^. FASN is a key enzyme in the de novo lipogenesis pathway and catalyzes the synthesis of palmitate from acetyl-CoA, malonyl-CoA and nicotinamide adenine dinucleotide phosphate (NADPH). High 2-HG in IDH1 MT tumors increases the consumption of NADPH and limits the availability of NADPH for de novo lipogenesis^11,12^, raising the possibility that such tumor cells could be selectively vulnerable to FASN inhibition in IDH1 MT tumors.

In this study, we show that IDH1 MT gliomas are sensitive to VPA treatment. We characterized the effect of VPA on the overall chromatin architecture of IDH1 MT gliomas and showed that VPA alters promoter accessibility and inhibits transcription of several lipogenic enzymes, including FASN, in IDH1 MT gliomas. We show that both VPA and the selective FASN inhibitor TVB-2640 rewire the lipidome and promote apoptosis in an IDH1 MT but not an IDH1 WT glioma cell line. These studies suggest that targeting fatty acid metabolism by HDAC and FASNi alone or in combination is a novel therapeutic opportunity for IDH1 MT gliomas.

## Results

### IDH1 MT glioma are sensitive to VPA in vitro and in vivo

**Fig. 1:**
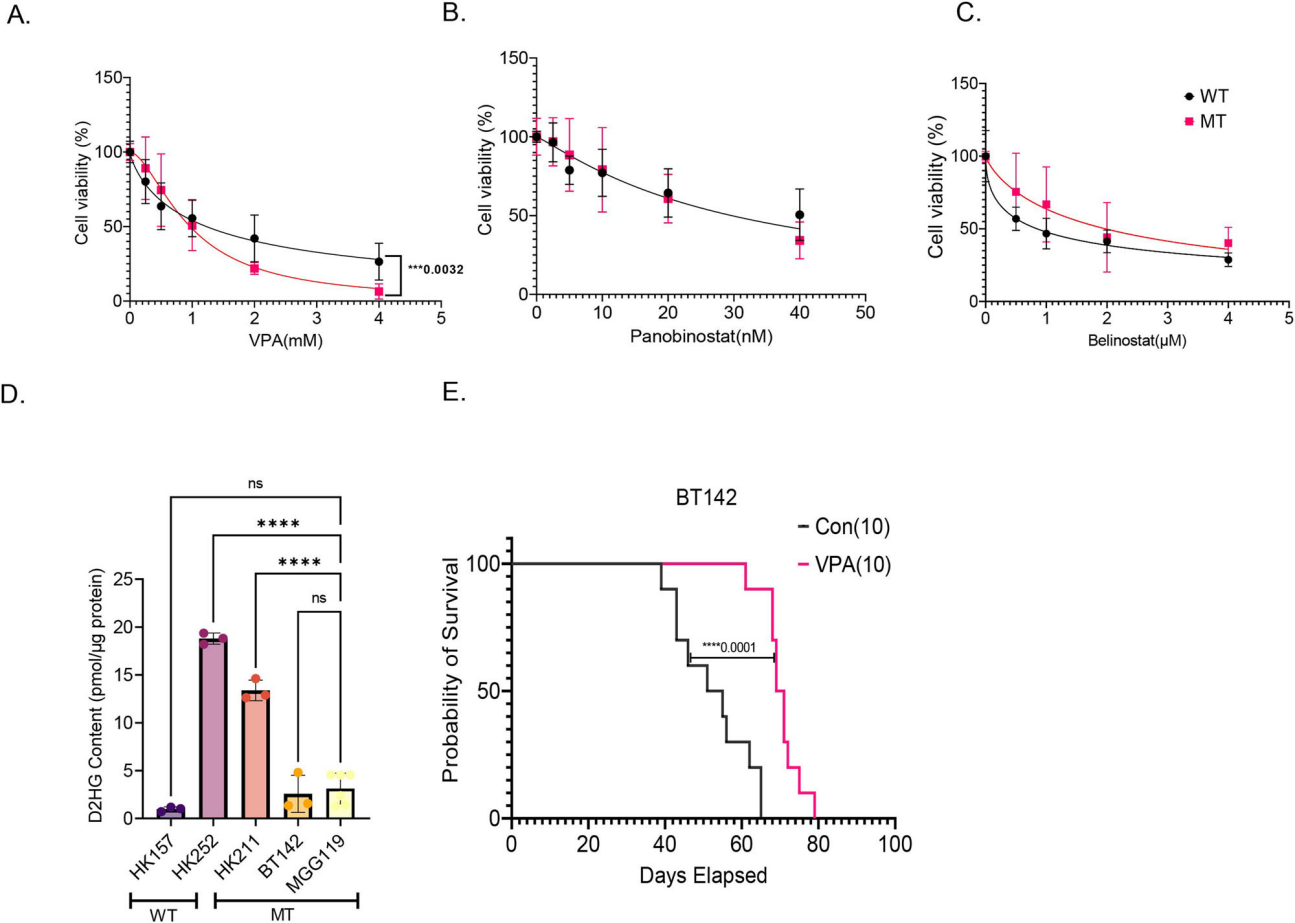
IDH1 MT are sensitive to VPA in vitro and in vivo. **A**–**C** Dose–response curve of IDH MT (3 cell lines) and IDH WT (2 cell lines) treated with VPA (**A**), Panobinostat (**B**), and Belinostat (**C**) for 1 week. **D** D-2HG content in the human primary WT & MT cell lines. **E** Kaplan–Meier survival curve for mice treated with either saline or VPA (300 mg/kg, twice daily) implanted with BT142. Non-linear regression, one-way ANOVA, ****P value<0.0001; ***P value<0.001; Error bars ± SD.

We tested three different HDACis, VPA, panobinostat, and belinostat in varying concentrations in IDH1 WT and IDH1 MT primary glioma cell lines. IDH1 MT glioma cell lines had a slightly lower IC50 for VPA compared to IDH1 WT cell lines (WTIC50 = 1.23; MTIC50 = 0.96), and the hill slope between the two dose–response curves were statistically significant (WTHill Slope = −0.80; MTHill Slope = −1.68; 0.0032) (Fig. 1A). Panobinostat (Fig. 1B) inhibited the growth of both IDH1 WT and IDH1 MT cell lines but there was no significant difference in the IC50 or hill slope between the two dose–response curves. The IDH1 WT cell lines tested were more sensitive to belinostat than IDH1 MT glioma cell lines (WTIC50 = 0.84; MTIC50 = 1.97) (Fig. 1C). As expected, the heterozygous IDH1 MT cell lines showed significantly higher 2HG levels compared to an IDH1 WT cell line (Fig. 1D). The 2HG content in hemizygous IDH1 MT cell line BT142 was approximately double that of the IDH1 WT cell line but the difference did not reach statistical significance. The heterozygous IDH1 MT cell lines HK252 and HK211 do not form xenograft in vivo, even in NSG mice; hence we tested the efficacy of VPA in vivo in the hemizygous BT142 model. Although BT142 has limited 2HG production (Fig. 1D), we found that the transcriptome of BT142 is most similar to our heterozygous IDH1 MT cell line HK252 and still distinct from IDH1 WT cell lines (Supplementary Fig. 1A), indicating that even with the loss of 2HG production, the line possesses many of the essential features of IDH1 mutant gliomas. We found that in vivo VPA treatment significantly improved the survival of mice in the BT142 xenograft model by 17 days (Fig. 1E).

**Fig. 2:**
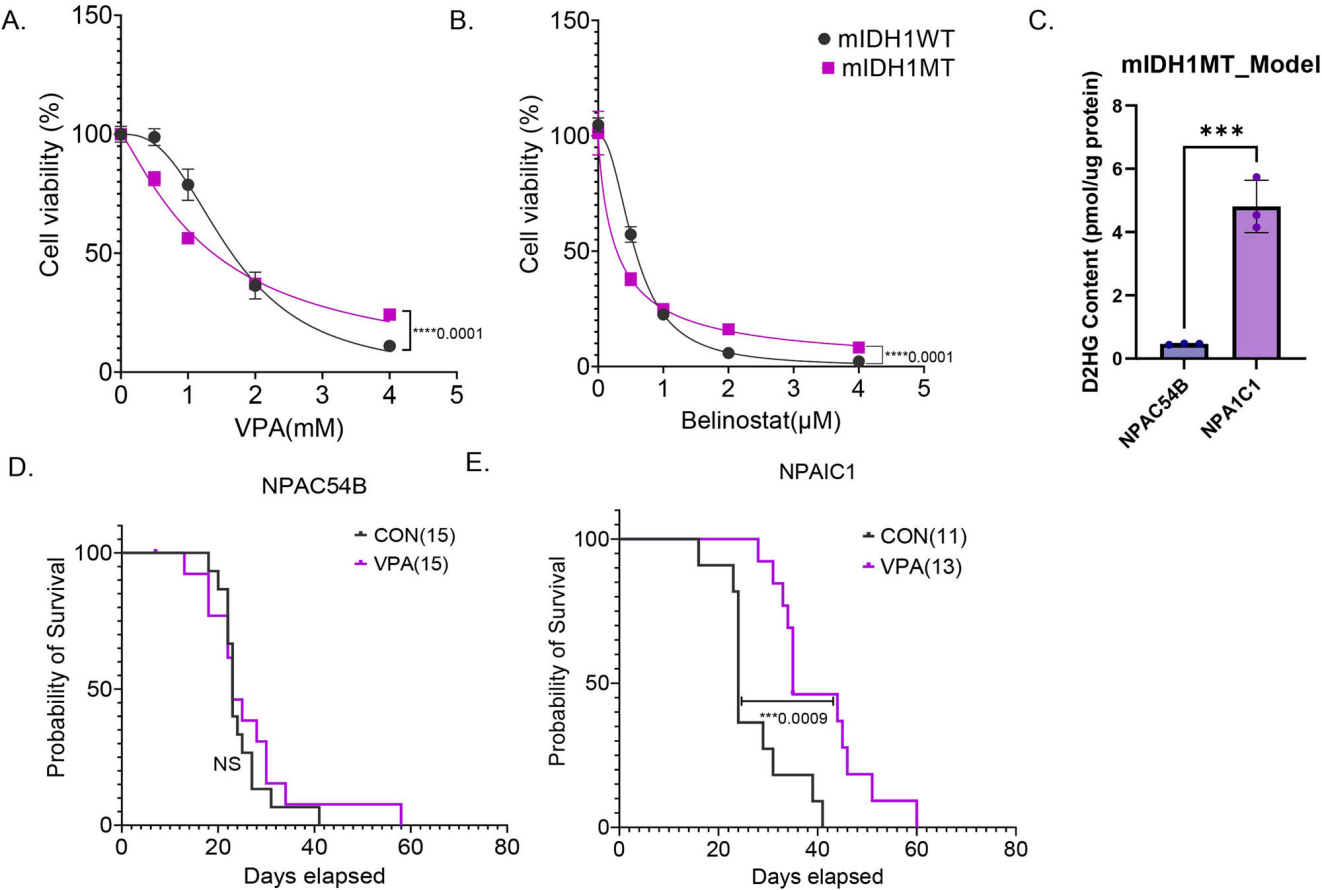
VPA improves survival of mice in a murine isogenic IDH MT model. **A** and **B** Dose–response curves of mIDH1MT and mIDH1WT treated with VPA (**D**) or Belinostat (**E**) for 5 days. **C** D-2HG content in murine mIDH1WT and mIDH1MT cell lines NPAC54B and NPAIC1. **D** and **E** Kaplan–Meier survival curve for mice with NPAC54B or NPAIC1 xenograft treated with either saline or VPA (300 mg/kg, twice daily. Non-linear regression, *T*-test, ****P value<0.0001; ***P value<0.001; Error bars ± SD.

Next, we tested the efficacy of HDACi’s in a syngeneic murine IDH1 (mIDH1) glioma model^13^. In vitro, NPAIC1(NRAS/IDH11^R132H^/shP53/shATRX) compared to mIDH1 WT cell line NPAC54B (NRAS/shP53/shATRX) had a slightly lower IC50 for both VPA (NPAC54BIC50 = 1.65; NPAIC1IC50 = 1.37) (Fig. 2A) and belinostat (NPAC54BIC50 = 0.57; NPAIC1IC50 = 0.28) (Fig. 2B). The mIDH1 MT cell line NPAIC1 also had significantly higher 2HG than mIDH1 WT cell line NPAC54B (Fig. 2C).

In vivo, VPA treatment did not improve the survival of mice bearing mIDH1 WT tumor xenografts (Fig. 2D) but significantly improved the survival of mice bearing mIDH1 MT tumor xenografts by 10 days (Fig. 2E). Thus, in the tumor models that we examined, we established that VPA diminished tumor growth in vivo and in vitro, but the murine model suggests a greater efficacy in IDH1 mutant tumors.

### VPA has opposing effects on overall gene expression and chromatin accessibility

**Fig. 3:**
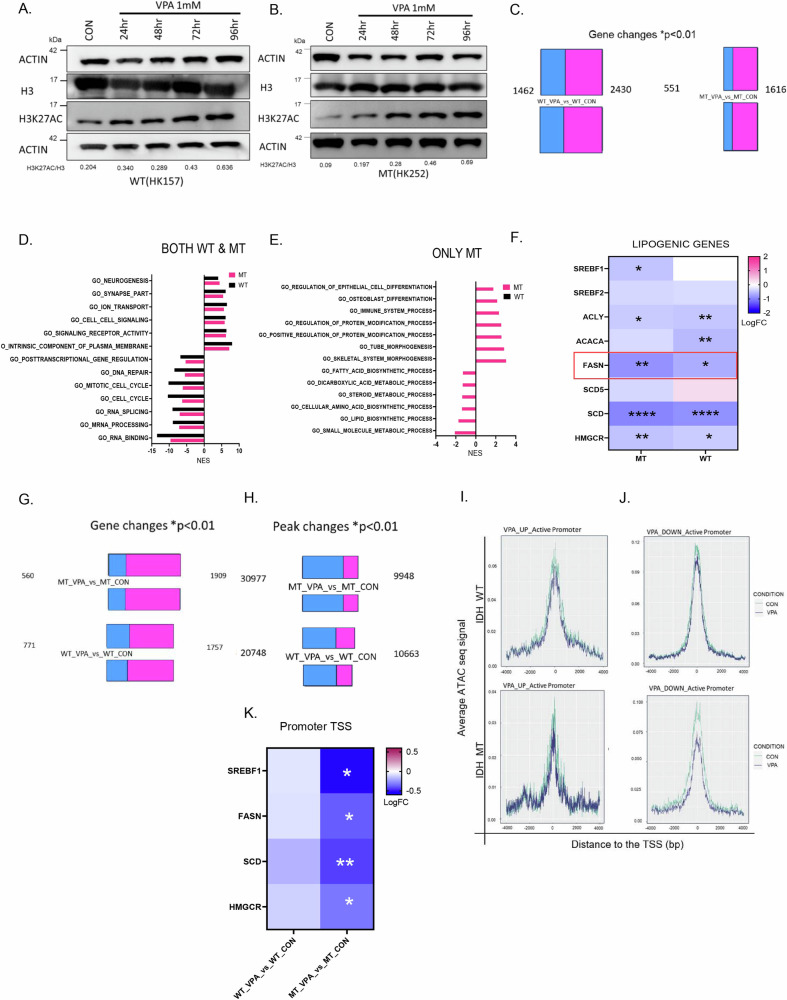
VPA decreases promoter chromatin accessibility and inhibits gene expression of lipogenic genes in IDH MT. **A** and **B** Representative western blot of an IDH WT cell line HK157 (A) and IDH MT cell line HK252 (**B**) showing an increase in H3K27ac over time with treatment of 1 mM VPA. **C** Differential gene expression changes, red (upregulated genes), green (downregulated genes) in IDH WT (5 cell lines) and IDH MT (4 cell lines) treated with 1 mM VPA for 48 h. **D** and **E** GSEA analysis shows top gene ontology terms upregulated and downregulated in both primary WT and MT cell lines (**D**) and only in MT but not WT (**E**). **F** Selected lipogenic genes that are downregulated in IDH WT and MT cell lines. **G** Differential gene expression changes, red (upregulated genes), green (downregulated genes) in IDH WT (2 cell lines) and IDH MT (3 cell lines) treated with 1 mM VPA for 5 days. **H** Differential peak changes, red (total number of ATAC peaks gained), green (total number of ATAC peaks lost) in IDH WT (2 cell lines) and IDH MT (3 cell lines) treated with 1 mM VPA for 5 days. **I** and **J** Active peak changes at the promoter (TSS=0) of VPA upregulated and downregulated genes in IDH WT (**I**) and IDH MT (**J**) GBM cell lines. **K** Selected lipogenic genes that show alteration in promoter chromatin accessibility in the MT but not WT cell lines. ****P value<0.0001; ***P value<0.001; **P value<0.01; *P value<0.05.

VPA is a multifaceted drug that has several direct^14^ and indirect targets^15^. Given the pleiotropic effects of VPA, we wanted to understand why IDH1 MT glioma cell lines showed some selectivity to VPA treatment. Given that HDACs are a known target of VPA, we treated an IDH1 MT cell line HK252 and an IDH1 WT cell line HK157 with 1 mM VPA and examined histone 3 lysine 27 acetylation (H3K27ac) over time. We found that after 96 h of treatment, VPA induced a strong increase in H3K27ac in both IDH1 WT (Fig. 3A) and IDH1 MT (Fig. 3B) cell lines, as would be expected with HDAC inhibition. We next treated multiple IDH1 MT glioma cell lines, HK252, HK213, BT142, & MGG119 (4) and IDH1 WT glioma cell lines, HK157, HK408, HK206, HK350, & HK393 (5) with 1 mM VPA for 48 h and conducted bulk RNA sequencing to get a better understanding of the transcriptomic effect of VPA. A greater number of genes were upregulated than downregulated with VPA treatment in both IDH1 WT and IDH1 MT glioma cell lines (Fig. 3C). The directionality of individual gene expression changes was similar for most WT and MT cell lines tested. However, we did notice some variability in the magnitude of individual gene expression changes among individual MT and WT cell lines tested. To better understand what biological processes are altered by VPA, we performed gene set enrichment analysis (GSEA) and compared the gene ontology (GO) terms that were up and downregulated with VPA treatment in IDH1 WT and IDH1 MT cell lines. We found in both IDH1 WT and IDH1 MT primary glioma cell lines, GO terms related to cell cycle and DNA repair were downregulated, whereas GO terms related to neurogenesis were upregulated after VPA treatment (Fig. 3D). We next asked what biological processes were only altered in IDH1 MT and not in IDH1 WT after VPA treatment. We found that VPA upregulated biological processes related to differentiation and downregulated lipid and sterol biosynthetic processes in the IDH1 MT but not IDH1 WT cell lines (Fig. 3E). We examined a panel of lipogenic genes and found that SREBF1, ACLY, FASN, SCD, and HMGCR which are all part of the de novo lipogenesis pathway were significantly downregulated in primary IDH1 MT cell lines after VPA treatment (Fig. 3F). In the IDH1 MT cell lines VPA inhibited expression of FASN and HMGCR more significantly than in the IDH1 WT cell lines. We also treated mIDH1WT and mIDH1MT with 1 mM VPA for 48 h and conducted RNA sequencing. We found that VPA downregulated FASN in the mIDH1MT but not in the mIDH1WT cell line (Supplementary Fig. 2A).

We next re-analyzed our previously published RNA-seq and ATAC-seq dataset^16^ of IDH1 MT and IDH1 WT cell lines treated with 1 mM VPA for 5 days and found that similar to the current findings, VPA treatment resulted in an increase in the expression of a greater number of genes and downregulated a smaller number of genes in both IDH1 WT and IDH1 MT cell lines (Fig. 3G). Interestingly, ATAC seq analysis showed that despite the relatively increased transcription, a greater number of peaks were lost, and only a few peaks were gained after VPA treatment (Fig. 3H), suggesting that VPA treatment activates transcription but paradoxically results in chromatin condensation. We next integrated ATAC and RNA seq data and when we examined the promoter region of VPA-upregulated genes in both IDH1 WT and IDH1 MT cell lines, we found that most genes did not show a change in promoter accessibility (Fig. 3I). There was a stronger correlation between promoter chromatin accessibility and VPA downregulated genes in IDH1 MT compared to the IDH1 WT cell lines (Fig. 3J). Interestingly, promoter accessibility of lipogenic genes was significantly altered in the IDH1 MT but not IDH1 WT cell lines (Fig. 3K). Taken together, our findings thus far indicate that VPA, while inhibiting the growth of both IDH1 mutant and wildtype cells, has different molecular effects on the IDH1 mutant and wildtype cells that we examined.

### The mTOR pathway is involved in the downregulation of FASN by VPA

The mechanistic target of rapamycin (mTOR) signaling pathway plays an important role in regulating lipogenesis^17^ and VPA has been found to inhibit the mTOR pathway in some cancer cell lines^18,19^, so we wondered whether VPA might be inhibiting lipogenic genes via the mTOR pathway. FASN is a key enzyme in the de novo lipogenesis pathway and although VPA targeted several enzymes involved in lipid metabolism, VPA inhibited transcription of FASN significantly more in the IDH1 MT compared to IDH1 WT. In addition, both in our IDH1 MT primary tumor cell lines and murine model, VPA inhibited transcription of FASN; hence we focused our attention on FASN.

**Fig. 4:**
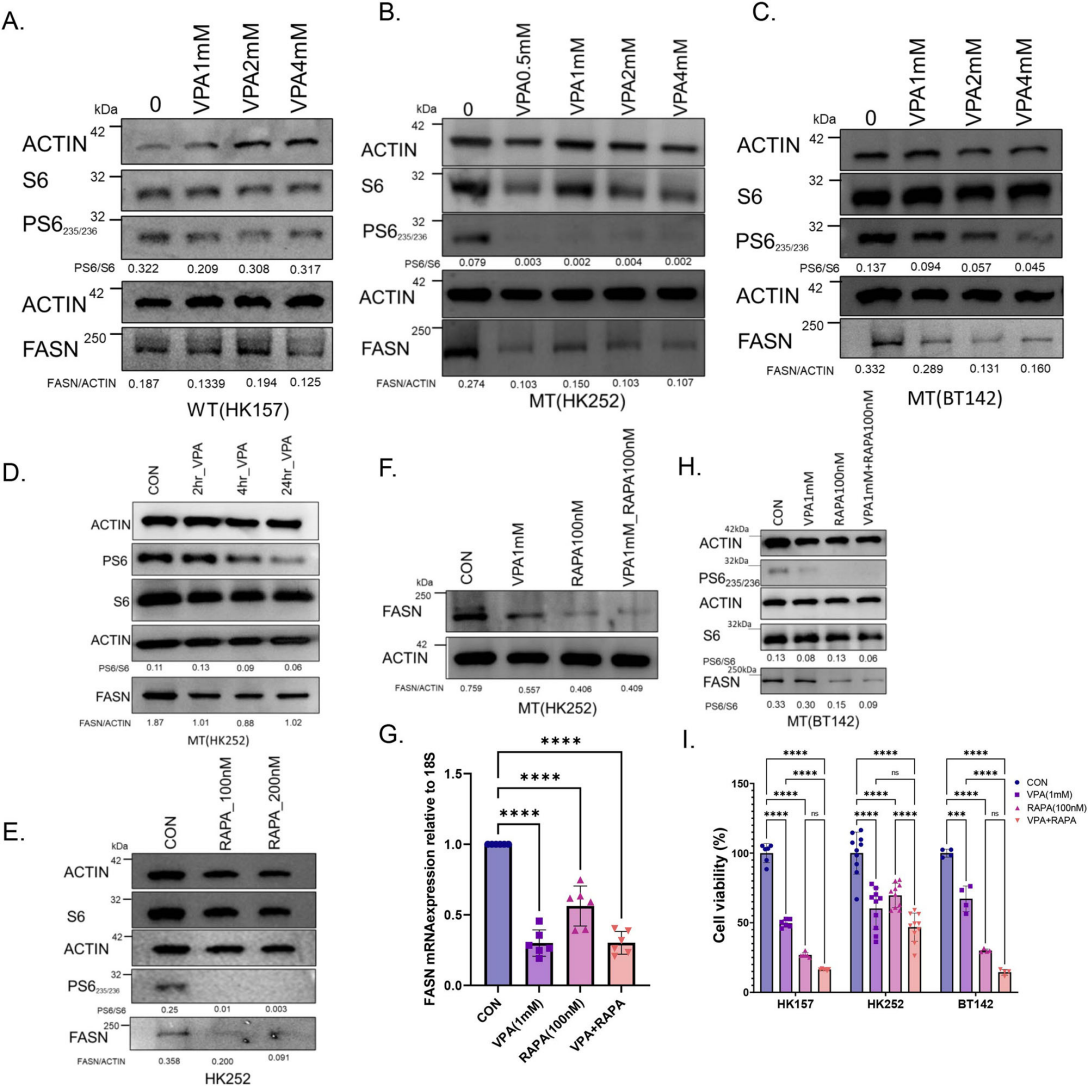
VPA may regulate FASN mRNA and protein through inhibition of mTOR signaling. **A**–**C** Representative western blots of PS6 and FASN in an IDHWT GBM cell line HK157 (**A**), and IDH MT GBM cell lines HK252 (**B**) and BT142 (**C**) treated with different doses of VPA for 4 days. **D** Representative western blot of PS6 and FASN in MT cell line HK252 treated with VPA for 2, 4, and 24 h. **E** Representative western blot of PS6 and FASN in MT cell line HK252 treated with rapamycin for 4 days. **F** Representative western blot of PS6 and FASN in MT cell line HK252 treated with VPA, rapamycin, & the combination for 4 days. **G** Relative mRNA expression of FASN in MT cell line HK252 treated with VPA, rapamycin, and the combination for 4 days. **H** Representative western blot of PS6 and FASN in MT cell line BT142 treated with VPA, rapamycin, and the combination for 4 days. **I** Relative cell viability after treatment with VPA, RAPA, & combination for 1 week. 2-way ANOVA and post hoc *t*-test ****P value<0.0001; ***P value<0.001; Error bars ± SD.

We treated IDH1 WT cell line HK157 and 2 IDH1 MT glioma cell lines, HK252 and BT142, with VPA for four days and examined phosphorylation of ribosomal protein S6 (PS6) and FASN protein expression. While VPA treatment had a minimal effect on PS6 and FASN protein expression in the IDH1 WT cell line HK157 (Fig. 4A) it resulted in diminished PS6 and FASN protein expression in IDH1 MT cell lines HK252 (Fig. 4B) and BT142 (Fig. 4C). Treatment of HK252 with VPA resulted in the reduction of pS6 within 24 h, possibly as early as 4 h (Fig. 4D). However, we note a reduction in FASN within 2 h. This suggests that the initial reduction of FASN expression is not directly downstream of mTOR inhibition. Treatment of the IDH1 MT cell line HK252 with rapamycin (RAPA) also diminished PS6 and FASN protein expression (Fig. 4E). This is consistent with previous literature that found that rapamycin treatment can contribute to reduced FASN expression^20^. In HK252, the combination of VPA with RAPA was not better than RAPA alone in decreasing FASN protein expression (Fig. 4F). RAPA treatment also inhibited FASN mRNA expression in HK252 while the addition of VPA to RAPA did not further diminish mRNA expression of FASN in HK252 (Fig. 4G). In BT142 VPA concentration greater than 1 mM was better at suppressing PS6 and FASN (Fig. 4C), and the combination of VPA1mM with RAPA100nM was better at suppressing FASN protein expression (Fig. 4H). Interestingly, the combination of VPA with RAPA was not better than VPA alone in inhibiting growth in HK252, but in HK157 and BT142, the combination of VPA and RAPA was better than VPA alone in inhibiting growth (Fig. 4I). This suggests that the combination of VPA with RAPA may be better in growth inhibition for some IDH1 MT cell lines due to better FASN suppression.

We also tested the effect of VPA and RAPA on mIDH1 WT and mIDH1MT cell lines. Surprisingly, VPA treatment resulted in increased PS6 in the mIDH1WT cell line but treatment diminished PS6 in the mIDH1 MT cell line in a dose-dependent manner (Supplementary Fig. 3A). Treatment with RAPA inhibited the growth of only mIDH1 MT but not mIDH1 WT. The combination of VPA and RAPA was better at inhibiting the growth of mIDH1 WT but not mIDH1 MT cell line (Supplementary Fig. 3B). In summary, we conclude that in both the IDH1 MT human primary tumor cell lines and in the murine model VPA inhibits growth at least partially via inhibition of the mTOR pathway and that the mTOR pathway is involved in downregulation of FASN. However, there is line-to-line heterogeneity in the relative extent to which FASN and mTOR signaling are connected.

### Inhibition of FASN inhibits the growth of IDH1 MT glioma cell lines

**Fig. 5:**
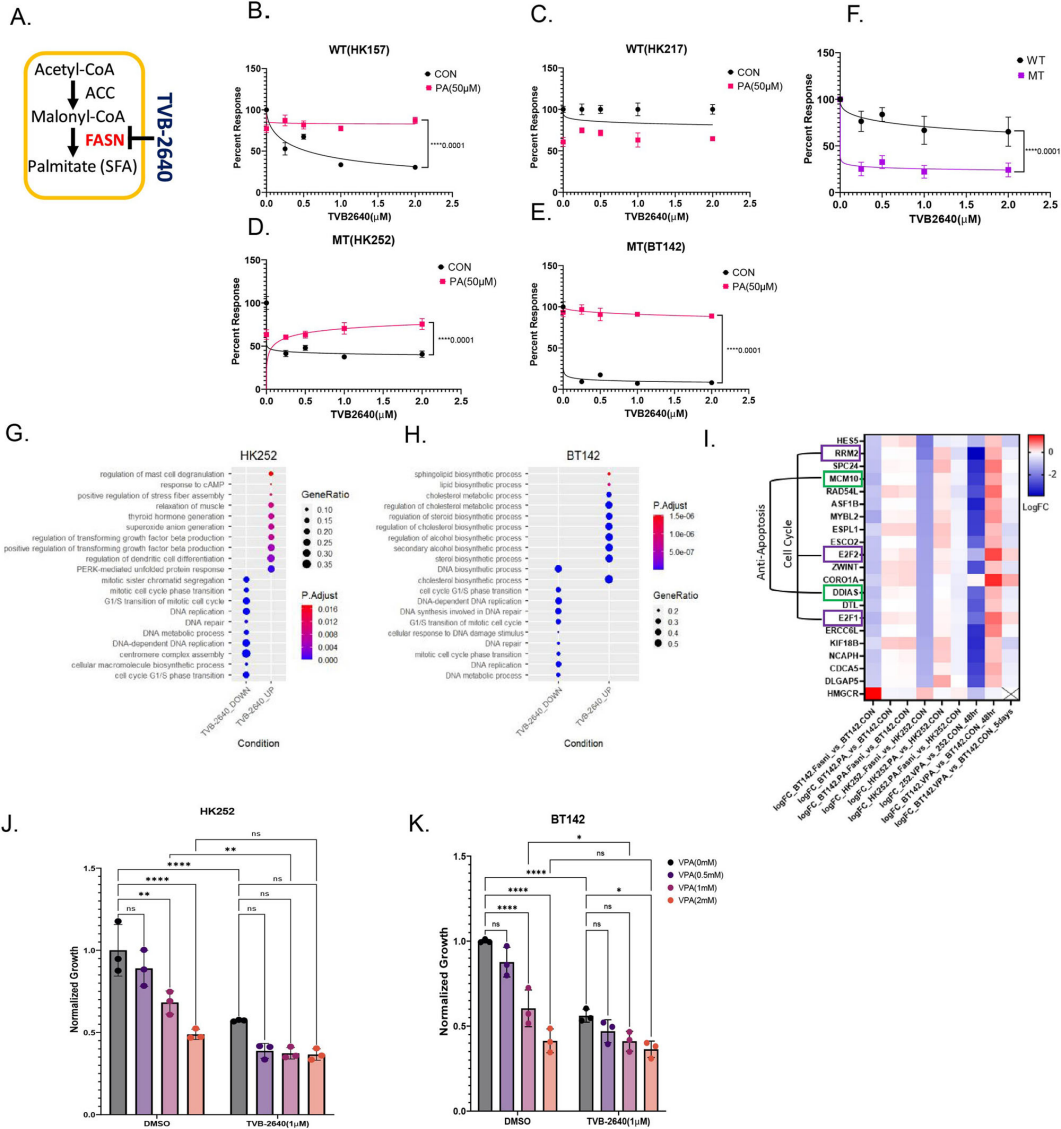
Selective FASN inhibitor TVB-2640 inhibits cell cycle genes in MT cell lines which can be recovered by supplementation of palmitate. **A** Schematic of TVB-2640 inhibiting FASN. **B**–**F** Relative cell viability of WT cell line HK157 (**B**) and 217 (**C**) and MT cell lines HK252 (**D**), BT142 (**E**) treated with TVB-2640 in the presence and absence of palmitate for 1 week. The group means ± SD are shown in **F**. Non-linear regression, ****P value<0.0001. **G** and **H** Dot plot showing upregulated and downregulated GO terms for MT cell lines HK252 (**B**) and BT142 (**C**) after 4 days of treatment with TVB-2640 (1 µM). **I** Heat map of common genes inhibited by TVB-2640 and VPA in both HK252 and BT142. **J** and **K** Relative growth of HK252 and BT142 treated with VPA (1 mM), TVB-2640 (1 µM), and PA (50 µM) for 1 week. 2-way ANOVA, post hoc *t*-test, ****P value < 0.0001; **P value < 0.01; **P value < 0.05.

Next, to test whether direct inhibition of FASN has any effect on the growth of IDH1 MT cell lines, we treated IDH1 WT and MT human gliomashpere cell lines with a selective FASN inhibitor (FASNi) TVB-2640 (Fig. 5A). We found that TVB-2640 inhibited the growth of WT cell line HK157 (Fig. 5B) but not HK217 (Fig. 5C) and inhibited growth of both MT cell lines HK252 (Fig. 5D) and BT142 (Fig. 5E). The MT cell lines tested on average were more sensitive to TVB-2640 than the WT cell lines tested (Fig. 5F). Supplementation of palmitate in the media rescued the growth inhibitory effect of TVB-2640 in both WT and MT cell lines, indicating that the negative effects on growth were likely mediated by FASN inhibition. In the murine model, TVB-2640 inhibited the growth of both mIDH1 WT and mIDH1 MT(Supplementary Fig. 4A). Thus, while FASN expression is more sensitive to VPA treatment in IDH1 mutant cells, both IDH1 MT and IDH1 WT cells are sensitive to direct FASN inhibition.

To better understand the effects of FASN inhibition, we treated IDH1 MT gliomasphere cultures with TVB-2640, palmitate, and the combination for 4 days and performed RNA sequencing. We found that TVB-2640 downregulated biological processes related to DNA repair and cell cycle in both HK252 (Fig. 5G) and BT142 (Fig. 5H). Genes related to cell cycle, such as (E2F1, E2F2, RRM2) and anti-apoptosis (DDIAS, MCM10), were downregulated by TVB-2640, and the expression of these genes could be recovered by palmitate supplementation (Fig. 5I). However, we noticed that TVB-2640 upregulated different biological processes in HK252 and BT142. In HK252, biological processes such as TGFβ production, stress fiber assembly and unfolded protein response were upregulated, whereas in BT142, sphingolipid and cholesterol biosynthetic processes were upregulated after FASN inhibition. When we examined individual genes, we found that 3-hydroxy-3-methylglutaryl-CoA reductase (HMGCR) was upregulated in both HK252 and BT142. Treatment with lovastatin, an HMGCR inhibitor, inhibited the growth of both MT cell lines HK252 and BT142 (Supplementary Fig. 5A). The combination of lovastatin with TVB-2640 was better at inhibiting growth of HK252 (Supplementary Fig. 5B). This suggests that IDH1 MT glioma cell lines perhaps increase cholesterol biosynthesis as a compensation for FASN inhibition^21^. When we compared genes that were targeted by both TVB-2640 and VPA we found a suite of genes such as E2F1, E2F2, RRM2, DDIAS, and MCM10 were downregulated by both drugs. In BT142, these genes decreased after 5 days of treatment. However, VPA inhibited the expression of HMGCR, while TVB-2640 upregulated HMGCR in IDH1 MT cell lines. Together, we conclude that in IDH1 MT glioma cell lines, VPA may work through FASN to inhibit cell cycle and anti-apoptotic genes. However, while VPA treatment inhibited transcription of several lipogenic genes, TVB-2640 treatment did not inhibit all lipogenic genes but upregulated some lipogenic genes such as HMGCR and ACACA, suggesting the net metabolic changes induced by VPA and TVB-2640 may be different.

We next assessed the combinatorial effect of TVB-2640 and VPA. We treated HK252 (Fig. 5J) and BT142 (Fig. 5K) with VPA and TVB-2640 and the combination of both drugs for 1 week. In HK252, the combination of VPA with TVB-2640 was not significantly better than TVB-2640 (1 µM) alone in inhibiting growth. In BT142, the combination of VPA2mM with TVB-2640 (1 µM) but not the combination of VPA (1 mM) with TVB-2640 (1 µM) was significantly better than TVB-2640 (1 µM) alone in inhibiting growth. The lack of additivity of the two drugs suggests that at least part of the effect of VPA is mediated via FASN.

### VPA and TVB-2640 have distinct effects on free fatty acids in an IDH1 MT glioma cell line

**Fig. 6:**
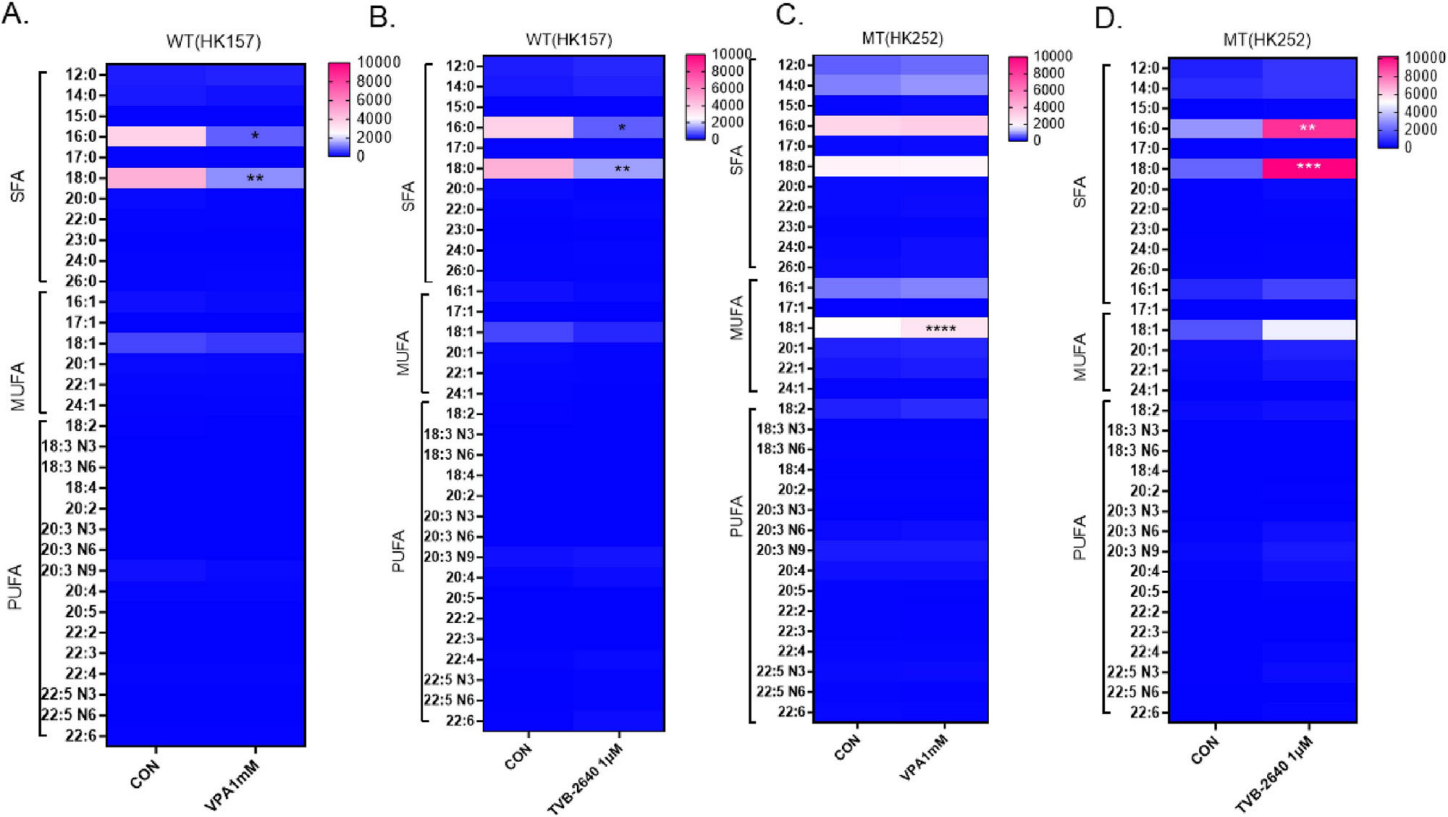
VPA and TVB-2640 increase free fatty acid in an IDH MT but not an IDH WT cell line. **A**–**D** Heatmap showing the change in saturated and unsaturated free fatty acids after 4 days of treatment with VPA and TVB-2640; 2-way ANOVA, post hoc *t*-test, ****P value < 0.0001; ***P value < 0.001; **P value < 0.01; *P value < 0.05.

The primary product of FASN is palmitate. Therefore, we next conducted lipidomics to measure palmitate and other free fatty acids after VPA orTVB-2640 treatment. In the IDH1 WT gliomasphere cell line, HK157, treatment with both VPA (Fig. 6A) and TVB-2640 (Fig. 6B) for 4 days significantly decreased free palmitic and stearic acid. Surprisingly, treatment with TVB-2640 significantly increased free palmitic and stearic acid in HK252 (Fig. 6C), suggesting the involvement of a compensatory mechanism or mechanisms. VPA treatment, on the other hand, significantly increased oleic acid but did not alter palmitic or stearic acid in the IDH1 MT cell line HK252 (Fig. 6D). This further suggests that while VPA targets FASN, the net metabolic effect of VPA and TVB-2640 on the lipidome is distinct in the IDH1 MT glioma cell line and it is different when compared to the IDH1 WT cell line HK157. In the IDH1 WT cell line HK157, both VPA and TVB-2640 decreased free saturated fatty acids, while in HK252 TVB-2640 significantly increased free saturated fatty acids. However, in HK252 VPA shifted the ratio of free fatty acids towards monounsaturated fatty acids, suggesting that VPA may not be solely working via inhibition of FASN.

### VPA and TVB-2640 alter lipid droplets and induce apoptosis

**Fig. 7:**
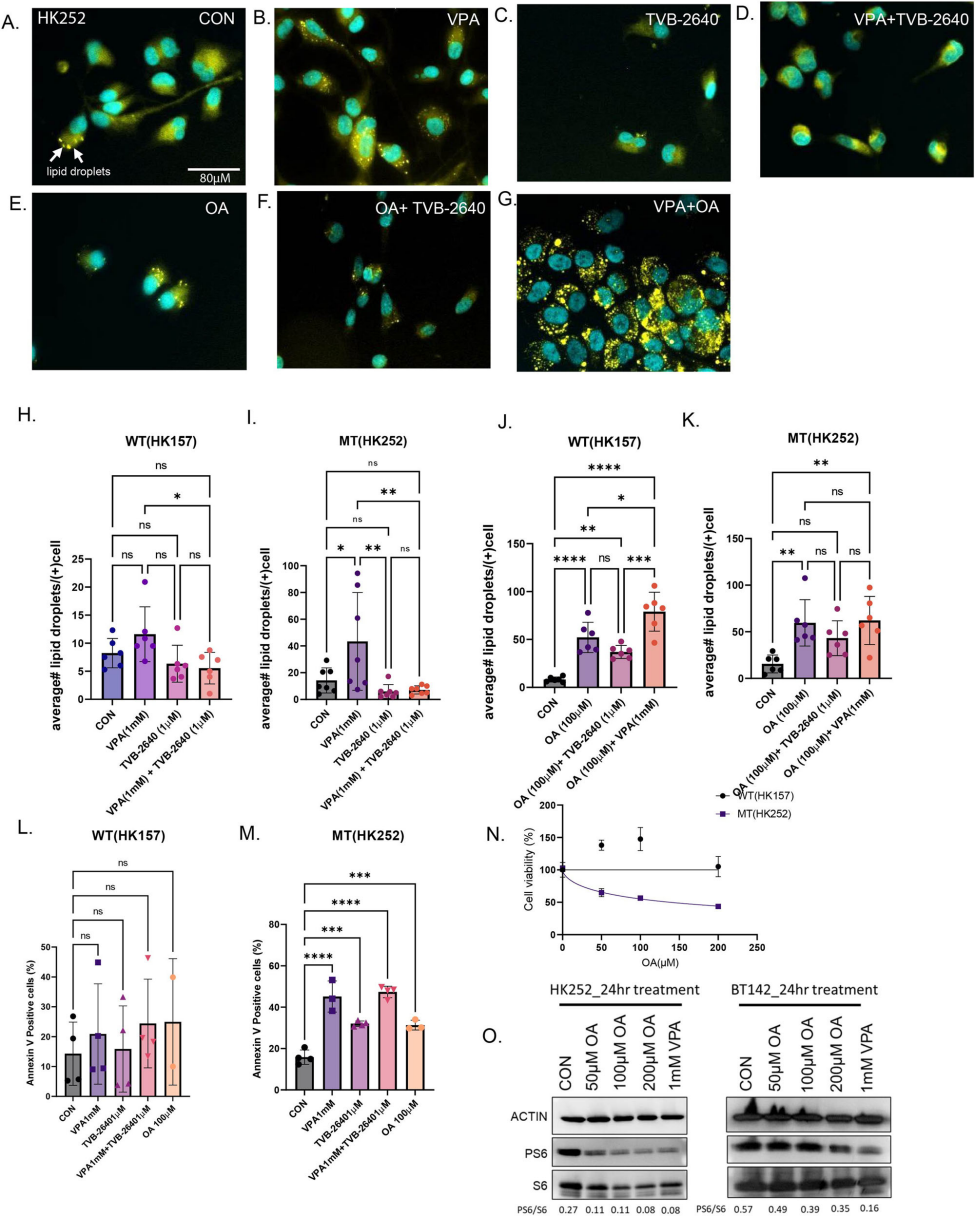
VPA and TVB-2640 alter lipid droplet formation and promote apoptosis. **A**–**G** Representative immunofluorescence images of lipid droplets in HK252 stained with Lipidspot^TM^. **H**–**K** Quantification of lipid droplets per cell in each condition. **L** and **M** Flow cytometry quantification of Annexin V positive cells in HK252 and HK157. **N** Cell viability of HK157 and HK252 after being treated with different concentrations of oleic acid for 1 week. Non-Linear Regression. **O** Representative western blot of PS6 in mutant cell lines HK252 and BT142 treated with OA and VPA for 24 h. 2-way ANOVA, post hoc *t*-test, ****P value < 0.0001; ***P value < 0.001; **P value < 0.01; **P value < 0.05; Error bars ± SD.

To prevent lipotoxicity, excess free fatty acids are often converted into neutral lipids and stored in organelles called lipid droplets. Both oleic acid^22^ and VPA^23^ have been previously found to induce lipid droplets. Our lipidomic data suggests that both VPA and TVB-2640 can increase free fatty acids in IDH1 MT, so we wondered if this might also have an effect on lipid droplet formation. In the IDH1 MT cell line HK252, treatment with VPA for 4 days increased the number of lipid droplets in lipid droplet-positive cells, as shown in Fig. 7B and quantified in Fig. 7I when compared to control (Fig. 7A, I). Treatment with TVB-2640 did not increase lipid droplet formation (Fig. 7C, I). This is probably because treatment with TVB-2640 alone does not significantly increase oleic acid, which is a lipid droplet inducer^22^. The combination of VPA with TVB-2640 inhibited the accumulation of lipid droplets (Fig. 7D, I), perhaps by preventing a significant increase in free oleic acid. Treatment of the IDH1 MT cell line HK252 with 100 µM oleic acid also increased lipid droplets (Fig. 7E, K), and TVB-2640 in combination with oleic acid (Fig. 7F, K) inhibited lipid droplet formation. The combination of VPA and oleic acid also increased lipid droplets compared to control (Fig. 7K). Lipid droplets in the VPA with oleic acid condition appeared much larger and appeared to fuse with each other (Fig. 7G). Interestingly, unlike HK252, where we found a significant increase in lipid droplets with both VPA (Fig. 7I) and oleic acid (Fig. 7K), in HK157, oleic acid significantly increased lipid droplets (Fig. 7J), but VPA did not (Fig. 7L). These findings indicate that the increase in lipid droplets in HK252 may be connected to the increase in free oleic acid induced by VPA. However, in HK157, we saw a decrease in free fatty acids, and hence we saw no change in lipid droplets.

We further quantified the amount of annexin V positive cells after VPA, TVB-2640, and oleic acid treatment and found that, compared to control, all three treatments did not increase the proportion of increased the proportion of annexin V positive cells in the IDH1 WT cell line, HK157 (Fig. 7L) but did in the only in the IDH1 MT cell line HK252 (Fig. 7M). Oleic acid treatment also inhibited the growth of IDH1 MT cell line HK252 but not WT cell line HK157 in a dose-dependent manner (Fig. 7N), indicating that one mechanism of cell death in VPA treatment could be lipotoxicity due to oleic acid. We also examined what fraction of cells that were positive for lipid droplets were also positive for annexin V by flow cytometry (Supplementary Fig. 6A). In MT cell line HK252, we found that after VPA treatment about 30% of the annexin V positive cells were also positive for lipid droplets whereas 70% of annexin V positive cells were not positive for lipid droplets (Supplementary Fig. 6B). This suggests that not all cells that are treated with VPA undergo lipotoxicity-induced apoptotic cell death. The formation of lipid droplets in a fraction of apoptotic cells (30%) may be a compensatory mechanism to protect cells from lipotoxicity, although one that is incomplete in that the cells still undergo apoptosis.

Interestingly, TVB-2640, in combination with VPA, prevented lipid droplet formation, suggesting there might be some therapeutic benefit of combining VPA with TVB-2640. Lastly, treatment with oleic acid inhibited PS6 (Fig. 7O) in IDH1 MT cell lines suggesting oleic acid may indirectly contribute to mTOR inhibition by VPA.

### HDACs are involved in the regulation of lipogenic gene expression

Next, given the pleiotropic effects of VPA and the lack of selectivity of other broad-spectrum HDACi for IDH1 mutant tumors, we wondered whether the effect of VPA on lipogenic genes is dependent or independent of HDAC inhibition. We re-analyzed RNA seq data from our recently published work^16^ and assessed the effect of HDAC knockdown on fatty acid metabolism genes. We found that knockdown of HDAC 2, 3, 4, and 9 increased the expression of lipogenic genes such as FASN, stearyl coA desaturase (SCD) and HMGCR. HDAC1 knockdown slightly decreased the expression of a few lipogenic enzymes but interestingly, only HDAC6 knockdown inhibited transcription of FASN. HDAC6 knockdown also inhibited transcription of other lipogenic enzymes such as ACACA, SCD and HMGCR but seemed to have a greater inhibitory effect on FASN (Supplementary Fig. 7A). Using 2 different CRISPR sgRNAs, we prospectively knocked down HDAC6 in the HK252 cell line. HDAC6 is a microtubule deacetylase, and as expected, HDAC6 knockdown increased tubulin acetylation (Supplementary Fig. 7B). Similarly, treatment of HK252 with VPA increased tubulin acetylation (Supplementary Fig. 7C) in a dose-dependent manner suggesting that VPA may mediate some of its effect on lipogenic genes through HDAC6. HDAC6 knockdown also inhibited the transcription of FASN (Supplementary Fig. 7D) and inhibited the growth of MT cell line HK252 but not the WT cell line HK157 (Supplementary Fig. 7E). Taken together, the data indicate that the effect on lipogenic genes by VPA may be mediated through HDAC6. However, the upregulation of some of the lipogenic enzymes after HDAC knockdown potentially suggests a mechanism by which tumor cells might compensate and why some HDAC knockdowns inhibit the growth of IDH1 MT glioma cells and others do not.

### FASN knockdown inhibits IDH1 mutant tumor growth in vivo and may enhance the effects of VPA treatment

**Fig. 8:**
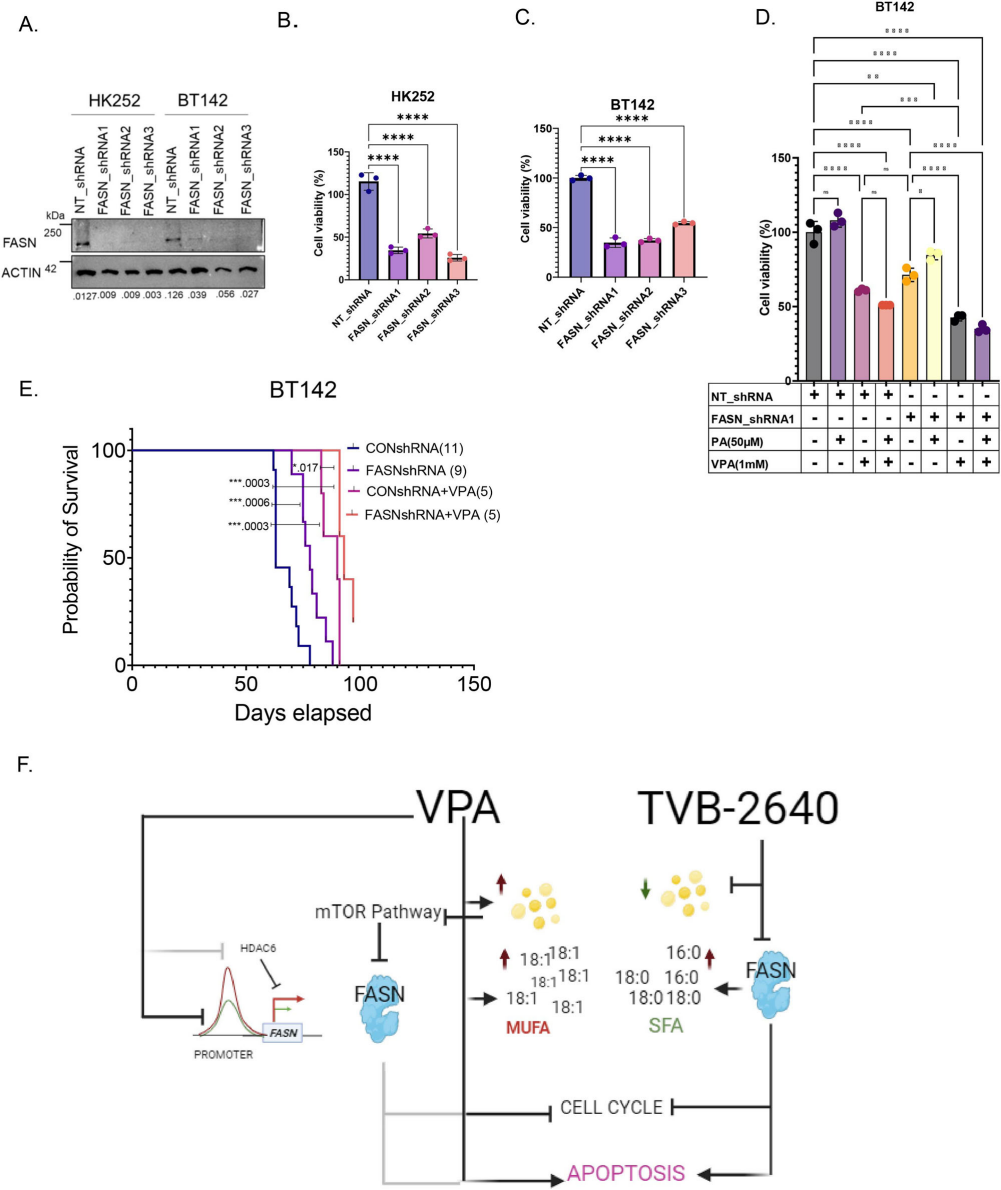
FASN knockdown enhances the effect of VPA in vivo. **A** Representative western blot showing FASN protein expression in HK252 and BT142 after FASN knockdown with three separate shRNAs. **B** and **C** Relative cell viability of MT cell lines HK252 (**B**) and BT142 (**C**) after FASN knockdown. One-Way ANOVA, ****P value <0.0001. **D** Relative growth of BT142 with ctrl non-target shRNA and FASN shRNA treated with VPA and PA for 1 week. **E** Kaplan-Meier survival curve for mice treated with either saline or VPA (300 mg/kg) implanted with NT BT142 or FASNshRNA1 KD BT142. ***P value < 0.001; *P value < 0.05. **F** Schematic of the mechanism of VPA and TVB-2640 in IDH MT glioma (created with bio render).

Because tumor cells may be able to compensate for the loss of FASN function in vivo through the utilization of exogenous fatty acids, it was critical for us to determine the effect of diminished FASN function in vivo. It is currently unclear whether TVB-2640 can cross the blood–brain barrier and the extent of off or on-target toxicities of the drug in mice. Hence, to test the effect of FASN inhibition in vivo we knocked down FASN in IDH1 MT lines BT142 and HK252 (Fig. 8A) using shRNAs. In vitro, FASN knockdown inhibited the growth of both HK252 (Fig. 8B) and BT142 (Fig. 8C). In BT142, palmitate rescued some of the growth inhibitory effect of FASN knockdown with FASN shrna1 (Fig. 8D). We further found that FASN knockdown in combination with VPA was better at inhibiting growth of BT142 when compared to VPA and FASN knockdown alone (Fig. 8D) in vitro. Interestingly, palmitate did not rescue the growth inhibitory of FASN knockdown in combination with VPA. Therefore, we further hypothesized that an in vivo combination of VPA with FASN knockdown may have a better growth inhibitory effect than either alone.

We found in vivo, compared to control, both VPA and FASN knockdown alone improved the survival of mice (Fig. 8E) in the BT142 model. Further, the combination of VPA with FASN knockdown slightly but significantly improved the survival of mice when compared to VPA or FASN knockdown alone (Fig. 8E). These findings indicate that in vivo, even in the presumed presence of exogenous palmitate, FASN knockdown alone can improve survival of mice. However, cancer cells can often use multiple ways to metabolically compensate or resist treatment; hence knockdown of FASN may improve the response to VPA in vivo.

## Discussion

In recent years several studies have emerged indicating that IDH1 MT gliomas are inhibited by broad-spectrum HDACis such as panobinostat^16,24^ and belinostat^25^. In our study, we found that IDH1 MT gliomas are more sensitive to VPA when compared to IDH1 WT glioma cell lines. In addition to in vitro studies, we show that VPA improved the survival of mice in vivo in two separate models of IDH1 MT glioma.

VPA is a drug with pleiotropic effects and our in-depth analysis suggests VPA has many targets and may ultimately work through multiple mechanisms to inhibit the growth of IDH1 MT gliomas but that at least some of the mechanistic effect is mediated by FASN as summarized in Fig. 8F. Consistent with its role as an HDACi, VPA increased histone acetylation and transcriptionally activated many more genes compared to downregulated genes in both IDH1 WT and MT glioma cell lines. Surprisingly, we found that although VPA increased transcription, instead of opening up the chromatin, it led to diminished chromatin accessibility, suggestive of increased chromatin condensation. Interestingly, a recent study using human primary cells showed VPA promoted both histone acetylation and deacetylation, and the increase in histone acetylation with VPA is regional^26^. The increase in gene expression with VPA that we observed in our glioma cell lines may be driven by alternate promoters^27^ or complex interaction between promoters and enhancers^28^.

We identified several metabolic genes, many of which are involved in the regulation of de novo lipogenesis and cholesterol metabolism to be downregulated by VPA treatment. ATAC seq data further showed that many of these metabolic genes lost chromatin accessibility at the promoters after VPA treatment. Although VPA decreased chromatin accessibility in both WT and MT cell lines, the loss of promoter chromatin accessibility for lipogenic genes was significant in IDH1 MT but not IDH1 WT glioma cell lines. Previously, the disruption of fatty acid metabolism has been found to decrease overall chromatin accessibility in the liver^29^; whether disruption of genes involved in lipogenesis also results in loss of chromatin accessibility is not known but we speculate that the disruption of lipogenic genes by VPA may contribute to the overall loss of chromatin accessibility seen in the IDH1 MT glioma cell lines.

We also found in both IDH1 MT glioma cell lines and a murine glioma model that VPA inhibited the mTOR pathway, and downregulated FASN mRNA and protein expression. The effect of VPA on the mTOR pathway seemed to be less prominent on the WT cell lines in both models. Previously, VPA has been shown to inhibit phosphorylation of mTOR, AKT, S6 and promote autophagy in glioma^18^, gastric^30^ and prostate cancer cell lines^19^. We found that treatment with rapamycin also inhibited FASN mRNA and protein expression in IDH1 MT glioma cell lines. This is consistent with the previous finding that showed that rapamycin targets lipid metabolism genes such as FASN^31^ and underscores that, at least in high-grade IDH1 mutant tumors, the mTOR pathway plays a significant role.

Our data showed that both the FASN inhibitor TVB-2640 and VPA downregulate a suite of cell cycle and anti-apoptotic genes. FASN inhibition has previously been found to induce both cell cycle arrest^32^ and apoptosis^33^ in tumor cells. Our RNA seq data suggested inhibition of cell cycle processes with VPA treatment, and we also saw an increase in annexin V-positive cells with VPA treatment, suggesting that VPA may exert its effect on both cell cycle and apoptosis via FASN downregulation. However, we cannot explain all the effects of VPA via FASN downregulation, as VPA also targeted other lipogenic enzymes such as SREBF1, SCD and HMGCR. Our data suggested that inhibition of HMGCR also inhibited the growth of IDH1 MT glioma cell lines, so it is possible that inhibition of both de novo lipogenesis and cholesterol metabolism contribute to the growth inhibitory effect by VPA.

Our lipidomic study suggested that although VPA targets FASN in IDH1 MT glioma cell lines, it does not deplete palmitate. In fact, direct inhibition of FASN by TVB-2640 significantly increased free palmitic acid in IDH1 MT cell line HK252. In breast cancer cells, FASN inhibition has been found to increase unsaturated fatty acids, ceramides, and diacylglycerols^34^. In prostate cancer cells, inhibition of FASN by multiple FASN inhibitors increased the synthesis of long-chain unsaturated fatty acids and phospholipids^35^. This suggests that cancer cells potentially upregulate multiple other metabolic pathways to compensate for FASN inhibition and this may be conserved among multiple cancer types. Unlike TVB-2640, VPA inhibited multiple metabolic enzymes which may be the reason why we didn’t observe a dramatic increase in palmitic acid with VPA treatment. Interestingly, In IDH1 WT cell line HK157, we saw a decrease in palmitic acid with both VPA and TVB-2640 treatment suggesting that VPA may also work through FASN in some IDH1 WT cell lines. However, it is important to note that although direct inhibition of FASN inhibits the growth of both WT and MT cell lines, the downstream lipidome rewiring in WT and MT cell lines is quite distinct.

The enzyme SCD is responsible for making oleic acid. Interestingly, we saw an increase in free oleic acid with VPA treatment in IDH1 MT cell line HK252, although VPA transcriptionally inhibited SCD. The increase in oleic acid could potentially be a compensatory mechanism, but even if it is, it is most likely a failed compensatory mechanism, as our data suggested that treatment with oleic acid-induced apoptosis and inhibited growth of the IDH1 MT glioma cell line. This is further supported by a recent study that showed that oleic acid shifted the balance towards monounsaturated fatty acids, causing apoptosis in IDH1 MT gliomas^36^. Although the prior work did not investigate lipid droplets, we found an increase in lipid droplets in the IDH1 MT glioma cell line with both VPA and oleic acid treatment. Cancer cells generally form lipid droplets to prevent ER stress and lipotoxicity^37^. The formation of lipid droplets in a fraction of apoptotic cells may be a compensatory mechanism to protect cells from lipotoxicity, although one that is incomplete in that the cells still undergo apoptosis.

HDACs are generally coupled to the expression of lipogenic enzymes in a complex manner. In fact, the knockdown of some HDACs in the IDH1 MT glioma cell line HK252 actually upregulated many lipogenic enzymes such as SCD and HMGCR. Our analysis showed a change in FASN mRNA expression only with HDAC6 knockdown in IDH1 MT glioma cell line HK252. VPA targets several HDACs and the fact that some HDAC knockdowns can upregulate metabolic genes is cautionary, as cancer cells can escape treatment through various mechanisms. It is possible that the effects of VPA on other HDACs influence lipid metabolism in different ways, contributing to the growth inhibitory or compensatory effects. For example, two prior studies identified HDAC3 to be responsible for deacetylation of FASN^10,38^. HDAC3 knockdown in the liver was also found to reroute precursors towards lipid synthesis and sequestration into lipid droplets^39^.

Lipogenic enzymes can be significant therapeutic targets in cancer, but it is important to note that cancer cells can use multiple metabolic pathways for survival. In our study, for instance, we found that only selective inhibition of FASN by TVB-2640 upregulated cholesterol metabolism in IDH1 MT gliomas. Cholesterol metabolism can compensate for FASN inhibition^21^. Interestingly, VPA targeted enzymes that are important for both lipogenesis and cholesterol metabolism. Perhaps VPA’s pleiotropic targets are more of an advantage than a disadvantage for the treatment of IDH1 MT gliomas. Nonetheless, we found that VPA increased lipid droplet formation in some cells, which might be a resistance mechanism. In vivo, the combination of VPA with FASN knockdown was also better than VPA and FASN knockdown alone in improving the survival of mice. Hence, it is possible that in order to overcome the metabolic flexibility of cancer cells, dual targeting of FASN by both VPA and TVB-2640 is potentially an effective therapeutic approach for IDH1 MT glioma.

### Study limitations

It is important to note that our human glioma models are focused on high-grade secondary gliomas, and not on low-grade gliomas. We do not yet know how treatment with VPA or other HDAC inhibitors will influence low-grade gliomas and how treatment with mIDH1-selective inhibitors will influence the effects of VPA. Furthermore, because of the limited number of cell lines studied, the differences between the responses in IDH mutant and WT cells observed here must be taken with some caution. There may be IDH WT tumors that are more “IDH mutant-like”, and that would respond to VPA in a similar manner. Lastly, we used hemizygous BT142 for our in vivo work which produces low 2HG. It has been reported that hemizygous IDH1 MT cell lines maintain many of the original epigenetic changes observed in original heterozygous cell lines^40^. In our analysis, BT142 clustered closely with other heterozygous IDH1 MT cell lines. Nonetheless, since we could do in vivo work with only a limited number of models it is possible that heterozygous primary IDH1 mutant cell lines may respond to VPA differently in vivo.

## Methods

All experiments in this study comply with relevant ethical regulations and have been approved by the UCLA Institutional Review Board. Protocols for animal studies were approved by the institutional animal care and use committee at UCLA. All patient samples were de-identified and collected with informed consent and with the approval of the UCLA Medical Institutional Review Board.

### Experimental cell lines and drugs

The primary patient-derived IDH1 WT and IDH1 MT cell lines were established in our laboratory and cultured in serum-free conditions as previously described^41^ with the exception of MGG119 which was a generous gift from Dr. Daniel Cahill. BT142 was purchased from ATCC. The murine glioma cell lines (NRAS G12V-shp53-shATRX-IDH11 wildtype; NPAC54B) and (NRAS G12V-R132H-shp53-shATRX-IDH11 mutant; NPAIC1) have been previously described^13^ and were cultured in the same media as patient-derived gliomaspheres. The cultures were regularly checked for mycoplasma and by STR to validate the source. The following drugs were used for the experiments. Valproic acid sodium salt,98% (Sigma-Aldrich, P4543-10G), panobinostat (Cayman, 13280), Belinostat (PXD101) (Sellechckem S1085), TVB-2640 (Sellechckem S9718), Oleic Acid (Cayman Chemical, 90260), Palmitic Acid (Sigma, 57-10-3), Rapamycin (LC laboratories, R5000).

### Animal studies

Studies did not discriminate sex, and both males and females were used. Strains: 8 to 12-week-old NOD-SCID gamma null (NSG) mice (NOD.Cg-*Prkdc*^*scid*^ *Il2rg*^*tm1Wjl*^/SzJ Jackson Laboratory, 00557) were used to generate tumors from a patient-derived glioma line BT142. C57BL6 (Jackson Laboratory, 000664) were used to transplant a murine NPAC54B and NPAIC1 as described previously^13^. Firefly-luciferase-GFP BT142 (1 × 10^5^) tumor cells were injected intracranially into the neostriatum of mice. 1 week after tumor cell line implant, mice were treated with VPA (300 mg/kg) of body weight twice a day every day of the week. For FASN knockdown and VPA experiment mice were treated with VPA (300 mg/kg) of body weight twice a day 5 days a week. Treatment continued until the animal reached the endpoint. Tumor growth was monitored once a week by measuring luciferase activity using IVIS Lumina II bioluminescence imaging. Survival: Mice were euthanized if they looked hunched, lost significant body weight, or showed fur changes and decreased activity than normal for survival analysis.

### Bulk RNA sequencing and analysis

Qiagen RNeasy microkit was used for RNA extraction from the gliomasphere. RNA quality was assessed using a Bioanalyzer and only samples with a RIN score > 8.0 were sequenced. RNA samples were pooled and barcoded, and libraries were prepared using TruSeq Stranded RNA (100 ng) + Ribozero Gold. Paired-end 2X75bp reads were aligned to the human reference genome (GRCh38.p3) using the STAR spliced read aligner (v 2.3.0e). Total counts of read fragments aligned to known gene regions within the human hg38 refSeq reference annotation were used as the basis for the quantification of gene expression. Differentially expressed genes were identified using EdgeR Bioconductor R-package, which are then considered and ranked based on false discovery rate (FDR Bejamini–Hochberg adjusted *p*-values of ≤0.01(not 0.1?)). Gene set enrichment analysis (GSEA) was carried out to determine the gene signatures differentially regulated in control and radiated cells and represented as heatmaps. R-Package V.3.2.5 (The R Project for Statistical Computing, https://www.r-project.org/) was used to generate the PCA plots and heatmaps. All sequencing datasets are available on GEO under the accession number GSE273405.

### ATAC sequencing and analysis

ATAC seq data was used from Garrett et al.^16^. Alignment of reads was carried out using the Burrows-Wheeler Aligner mem using hg19 assembly. Peak calling was performed using MACS2 (with parameter setting –nomodel –shift 75), and differential peak analysis using featureCount and DESeq2 (default setting). Motif analysis and peak annotation were done using HOMER and GO analysis using HOMER (http://homer.ucsd.edu/homer/) and EnrichR (https://maayanlab.cloud/Enrichr/). UCSC Genome Browser was used to determine whether open regions displayed H3K27Ac and conserved TF binding sites. Integrated Genome viewer (IGV) was used to represent the peaks/open regions.

### Free fatty acid panel GC–MS and analysis

1 × 10^6^ million cells per sample per condition in triplicate were submitted to UCSD Lipidomic Core. Samples were prepared using a previously published protocol by Quehenberger et al.^42^ and analyzed at UCSD Lipidomics Core.

### Cell viability assay

WT and MT cells were plated at a density of 5000 cells per well in 96-well plates. Proliferation was assessed 7 days after treatment with respective drugs using CellTiter-Glo® luminescent Cell Viability Assay (Fisher Scientific, PRG9242). The luminescence signal was measured in a luminometer, on day 0 and day 7. The luminescence signal was normalized to the initial reading at day 0.

### Western blot assay

At experimental endpoints cells were collected and lysed in RIPA buffer with protease and phosphatase inhibitors. Cells were kept on ice for 10 min and centrifuged at full speed for 5 min. Protein concentration was measured by Bradford assay using a BSA standard. Protein lysates were run on an SDS–PAGE on 4–12% gradient polyacrylamide gel (Thermo Fischer Scientific) and transferred onto nitrocellulose membranes. Blots were incubated with primary antibody diluted in 5% milk overnight. Blots were washed and incubated with HRP-conjugated secondary antibodies. Quantitation of protein levels was performed in ImageJ by normalizing to the loading control, β-actin. Antibodies FASN (CST, 3180), PS6235/236 (CST, 2211), S6 (CST, 2217), BACTIN (CST, 4970), HDAC6 (CST, 7558), H3K27AC (CST, 8173), H3 (CST 9717).

### Annexin V/7ADD assay

Cells were treated for 4 days with VPA and TVB-2640, and cells were prepared according to the manufacturer’s instruction for Biotium Annexin V and 7-AAD Apoptosis Kit and acquired by flow cytometry within an hour.

### Intracellular D-2-HG quantification

Intracellular D-2-HG was quantified using enzymatic assays originally described by Balss et al.^43^, who we thank for generously providing key reagents that are also commercially available in the D-2-HG Assay Kit (*MilliporeSigma*, Catalog #MAK320, Burlington, MA, USA). All experiments were performed in triplicate unless otherwise specified. Briefly, cells were harvested and lysed prior to splitting each sample into two aliquots for D-2-HG quantification and total protein quantification using the Pierce BCA Protein Assay Kit (*Thermo Fisher Scientific*, Catalog #23225, Waltham, MA, USA), respectively. Deproteination of the D-2-HG quantification aliquot was achieved using 3.0 mL of Proteinase K (*Qiagen*, Catalog #19131, Hildren, Germany) per 100.0 mL of cell lysis solution. 25.0 mL of lysate was then added to 75.0 mL of assay solution containing 0.1 mg of the enzyme D-2-HG dehydrogenase (HGDH), 100.0 mM NAD+, 5.0 mM resazurin, and 0.01 U/mL diaphorase in 100.0 mM HEPES (pH 8.0). In the presence of D-2-HG, HGDH converts D-2-HG to α-ketoglutarate (α-KG) in a NAD+-dependent manner. The reduction of NAD+ to NADH enables the conversion of resazurin to fluorescent resorufin, which was then quantified via fluorometric detection (*λ*_ex_ = 540 nm, *λ*_em_ = 590 nm) on a Wallace Victor2 1420 Multilabel HTS Counter (Perkin Elmer, Waltham, MA, USA). D-2-HG quantification (pmole/mg protein) of a given sample was based on a standard curve of known D-2-HG concentrations. Two-tailed Student’s *t*-tests were used to compare means between groups, with statistical significance set at *P* < 0.05.

### Lipid droplet staining and flow cytometry

LipidSpot Lipid Droplet Stain 647 was used to stain lipid droplets in gliomasphere cultures. Cells were plated on a 24-well dish in Cultrex UltiMatrix-covered coverslips. Cells were treated with drugs for 4 days, and at the experimental endpoint, media was removed, cells were washed, and incubated with 1X dye diluted in DPBS for 15 min at 37 °C. After 15 min the dye was removed, and cells were fixed with 4% PFA. Nuclei were stained with Hoechst and mounted on coverslips with vector shields. Coverslips were imaged and analyzed using Image J.

For flow cytometry quantification at the experimental endpoint, media was removed and cells were incubated with 1X dye diluted in DPBS for 30 min at 37 °C. After 30 min cells, the dye was removed, and cells were dissociated into single cells and acquired using flow cytometry.

### shRNA and CRISPR/Cas9 lentiviral knockdown

The following plasmids were used for knockdown experiments Scramble shRNA plasmid # 1864 (Addgene); FASN shRNA1 plasmid # 82327(Addgene); FASN shRNA2 _ TRCN0000002125 (Horizon); FASN shRNA2 _ TRCN0000002126 (Horizon); FASN_shRNA3_TRCN0000002127 (Horizon); HDAC6 sgRNA 5488: CAAGGAGCAACTGATCCAGG, HDAC6 sgRNA 5501: CCTAGATCGCTGCGTGTCCT, AAVS1 sgRNA: GGGCCACTAGGGACAGGAT were cloned into pLentiCRISPRv2. lentiCRISPR v2 was a gift from Feng Zhang (Addgene plasmid # 52961; http://n2t.net/addgene:52961; RRID: Addgene_52961)^44^.

These plasmids were transfected into 293T cells along with a 2nd-generation viral DR8.74 package and VSV-g envelope for the production of lentivirus. Cells were infected with lentivirus and puro selected for a week before doing growth experiments. For cells infected with FASN, shRNA media was supplemented with palmitic acid (50 µM) along with puromycin to prevent all knockout cells from dying.

### Statistical analysis

All data are expressed as mean ± SD. *P* values < 0.05 were taken as significant and were calculated in Graph Pad Prism 9.0 using one-way ANOVA for multiple comparisons with Bonferroni correction, followed by a post-hoc *t*-test. Log-rank analysis was used to determine the statistical significance of Kaplan–Meier survival curves. R-package was used for statistical analysis of sequencing experiments. Schematics used in the figures were generated in Biorender.

## Supplementary information

Supplementary information

## Data Availability

All sequencing datasets are available on GEO under the accession number GSE273405.
