# Publisher Correction: Valproic acid targets IDH1 mutants through alteration of lipid metabolism

**DOI:** 10.1038/s44324-024-00035-0

**Published:** 2024-10-18

**Authors:** Lubayna S. Elahi, Michael C. Condro, Riki Kawaguchi, Yue Qin, Alvaro G. Alvarado, Brandon Gruender, Haocheng Qi, Tie Li, Albert Lai, Maria G. Castro, Pedro R. Lowenstein, Matthew C. Garrett, Harley I. Kornblum

**Affiliations:** 1 Department of Psychiatry and Behavioral Sciences and the UCLA Intellectual and Developmental Disabilities Research Center, David Geffen School of Medicine, UCLA, Los Angeles, CA USA; 2 Department of Neurology, David Geffen School of Medicine, UCLA, Los Angeles, CA USA; 3 Department of Neurosurgery, Department of Cell and Developmental Biology, and Rogel Cancer Center, University of Michigan Medical School, Ann Arbor, MI USA; 4 Kettering Health Network, Kettering, OH USA; 5 Department of Molecular and Medical Pharmacology, David Geffen School of Medicine, UCLA, Los Angeles, CA USA

**Keywords:** Cancer metabolism, Oncology

Correction to: *npj Metabolic Health and Disease* 10.1038/s44324-024-00021-6, published online 13 August 2024

GEO accession number GSE273405 was mistakenly left out to the Data availability statement when this Article was first published. This has now been corrected in both the HTML and PDF versions of the Article.

