# Supplementary material for: Valproic acid targets IDH1 mutants through alteration of lipid metabolism
Source: NPJ Metab Health Dis. 2024 Aug 13;2:20. doi: 10.1038/s44324-024-00021-6 (PMC11321993; doi:10.1038/s44324-024-00021-6)
Supplement: Supplementary file 1 — Supplementary information [file 44324_2024_21_MOESM1_ESM.pdf]

A.

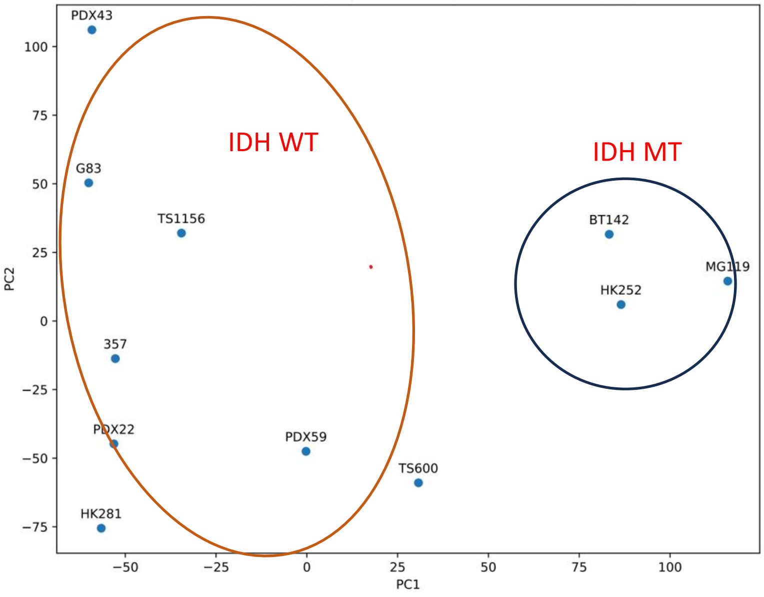
PDX43
G83
TS1156
357
PDX22
PDX59
TS600
HK281
BT142
MG119
HK252
IDH WT
IDH MT
100
75
50
25
0
−25
−50
−75
PC2
−50
−25
0
25
50
75
100
PC1

SFig1. The transcriptome of BT142 is similar to heterozygous IDH MT cell line and distinct from IDH WT cell lines.

A. Principal component analysis of bulk RNA sequencing of IDH MT and IDH WT cell lines. HK252, BT142 and MG119 are IDH MT cell lines and clusters together separately from all the IDH WT cell lines.

A.

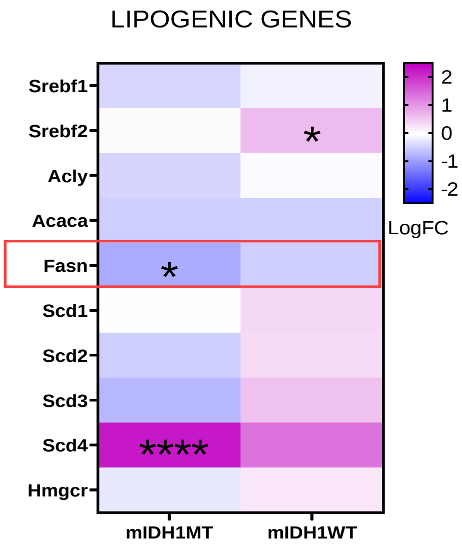
LIPOGENIC GENES
Srebf1
Srebf2
Acly
Acaca
Fasn
Scd1
Scd2
Scd3
Scd4
Hmgcr
*
*
****
mIDH1MT
mIDH1WT
2
1
0
-1
-2
LogFC

SFig2. VPA alters lipogenic gene expression in murine isogenic model of IDH MT.

A. Selected lipogenic genes that are differentially regulated in mIDH1WT and mIDH1MT cell lines. ****P value<0.0001; *P value<0.05;

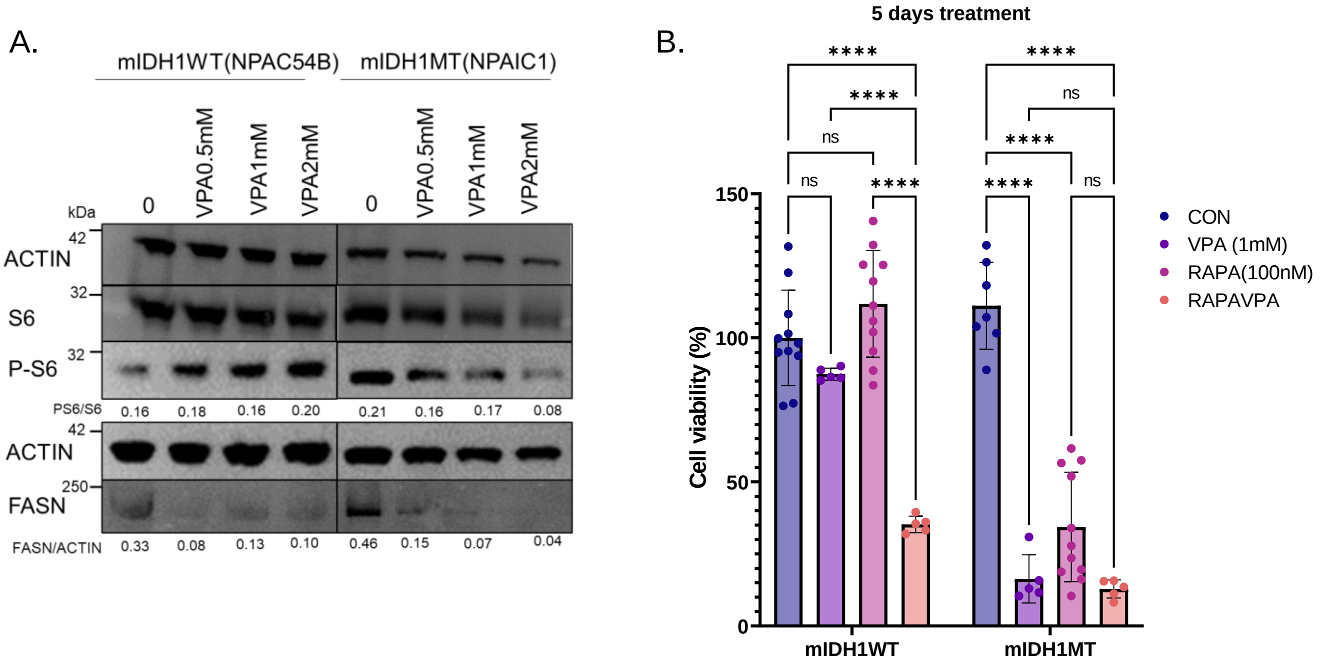
A.
mIDH1WT(NPAC54B)
mIDH1MT(NPAIC1)
kDa
0
VPA0.5mM
VPA1mM
VPA2mM
0
VPA0.5mM
VPA1mM
VPA2mM
42
ACTIN
32
S6
32
P-S6
PS6/S6 0.16 0.18 0.16 0.20 0.21 0.16 0.17 0.08
42
ACTIN
250
FASN
FASN/ACTIN 0.33 0.08 0.13 0.10 0.46 0.15 0.07 0.04
B.
5 days treatment
****
****
ns
ns
****
****
ns
****
****
ns
150
100
50
0
Cell viability (%)
mIDH1WT
mIDH1MT
CON
VPA (1mM)
RAPA(100nM)
RAPAVPA

SFig3: VPA decreases PS6 and FASN protein expression in mIDH1MT (NPAIC1) cell line.

A. Representative western blot of PS6 and FASN in NPAC54B and NPAIC1 treated with increasing concentration of VPA for 4 days.

B. Relative cell viability after treatment with VPA, RAPA, & combination for 5 days. 2-way ANOVA, post hoc t-test,****P value<0.0001; Results are expressed as the mean ±Standard deviation with individual datapoints illustrated.

A.

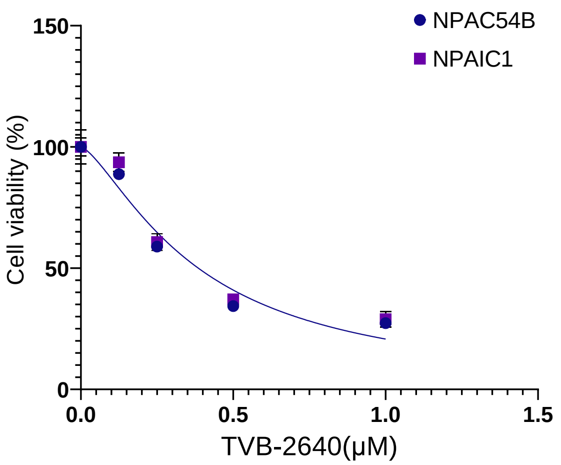

NPAC54B
NPAIC1
150
100
50
0
Cell viability (%)
0.0
0.5
1.0
1.5
TVB-2640(μM)

SFig4: Both NPAIC1 and NPAC54B respond to TVB-2640.

A. Dose response curve for NPAIC1 and NPAC54B treated with TVB-2640 for 4 days. The dot represents the mean at each respective dose. Error bars ±SD.

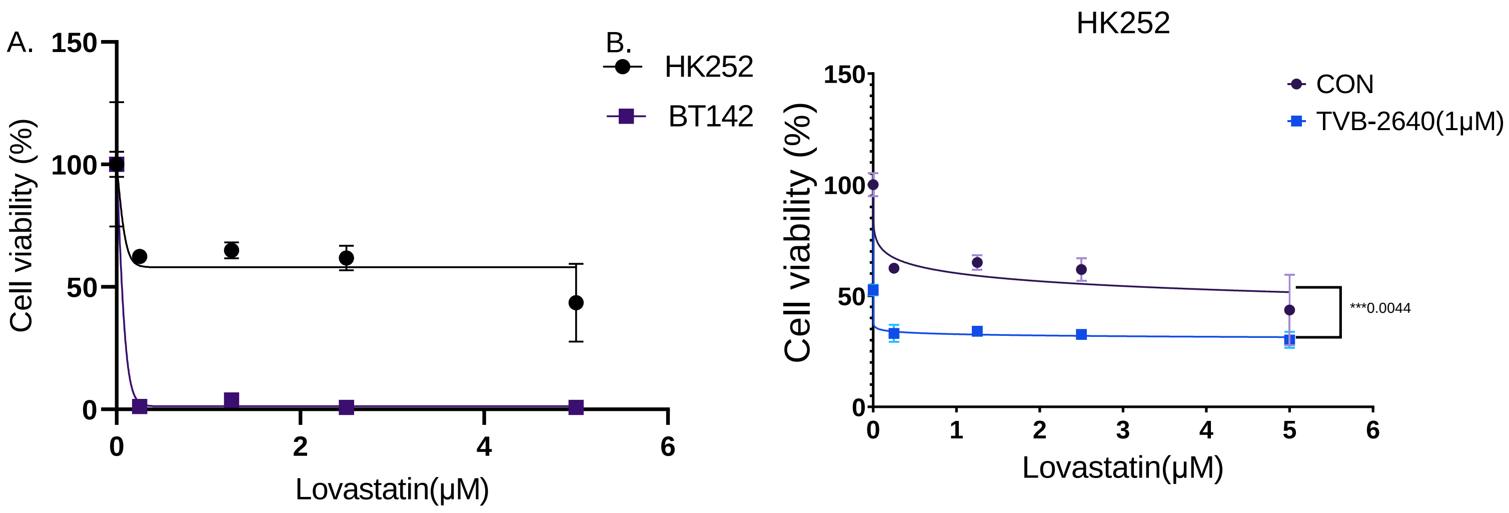
A.
B.
HK252
BT142
Cell viability (%)
Lovastatin(μM)
HK252
CON
TVB-2640(1μM)
Cell viability (%)
Lovastatin(μM)
***0.0044

SFig5: Lovastatin inhibits growth of IDH MT cell lines.

A. Dose response curve of HK252 and BT142 treated with lovastatin for 1 week. B. Dose response curve of HK252 treated with varying concentration of lovastatin in combination with 1µM of TVB-2640. The dot represents the mean at each respective dose. Non-linear regression. ***P value<0.001 Error bars ±SD.

A.

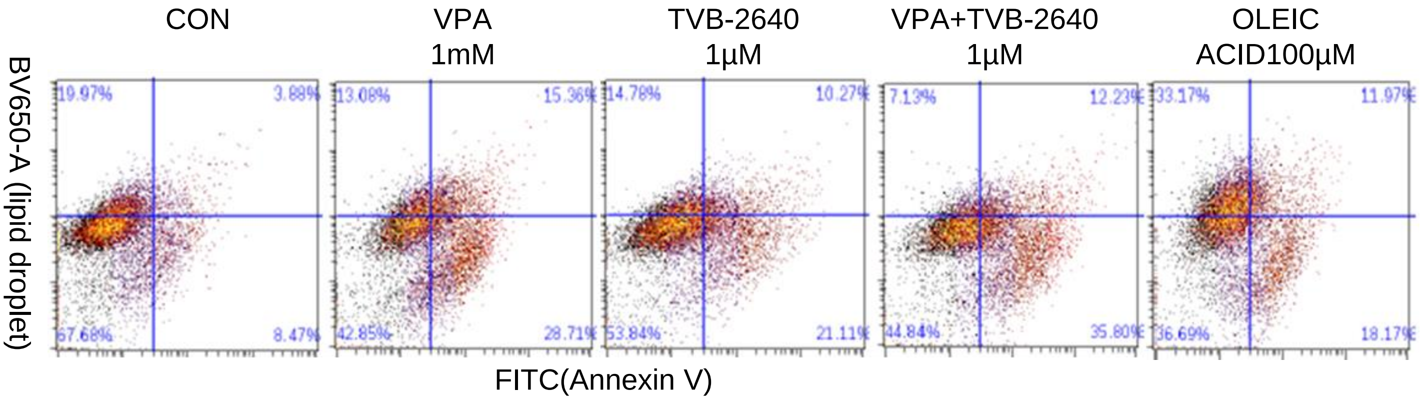
CON
VPA
1mM
TVB-2640
1µM
VPA+TVB-2640
1µM
OLEIC
ACID100µM
BV650-A (lipid droplet)
19.97%
3.88%
67.68%
8.47%
13.08%
15.36%
42.85%
28.71%
14.78%
10.27%
53.84%
21.11%
7.13%
12.23%
44.84%
35.80%
33.17%
11.97%
36.69%
18.17%
FITC(Annexin V)

B.

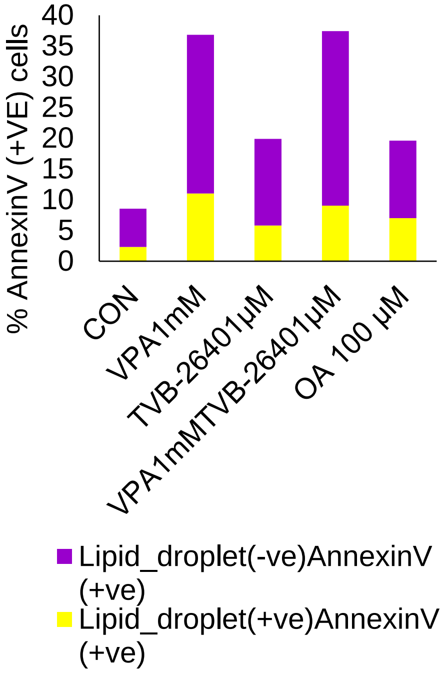
% AnnexinV (+VE) cells
0
5
10
15
20
25
30
35
40
CON
VPA1mM
TVB-26401µM
VPA1mMTVB-26401µM
OA 100 µM
Lipid_droplet(-ve)AnnexinV (+ve)
Lipid_droplet(+ve)AnnexinV (+ve)

SFig6: Relative proportion of cells that are positive for both lipid droplet and annexin V.

A. Representative flow cytometry traces of cells stained with Annexin V and Lipid droplets. Cells were treated for 4 days.

B. Quantification of proportion of annexin V and lipid droplet double positive cells. Purple bars indicate the percentage of lipid droplet negative cells that are Annexin V positive, yellow indicates the percentage of lipid droplet positive cells that are also Annexin V positive

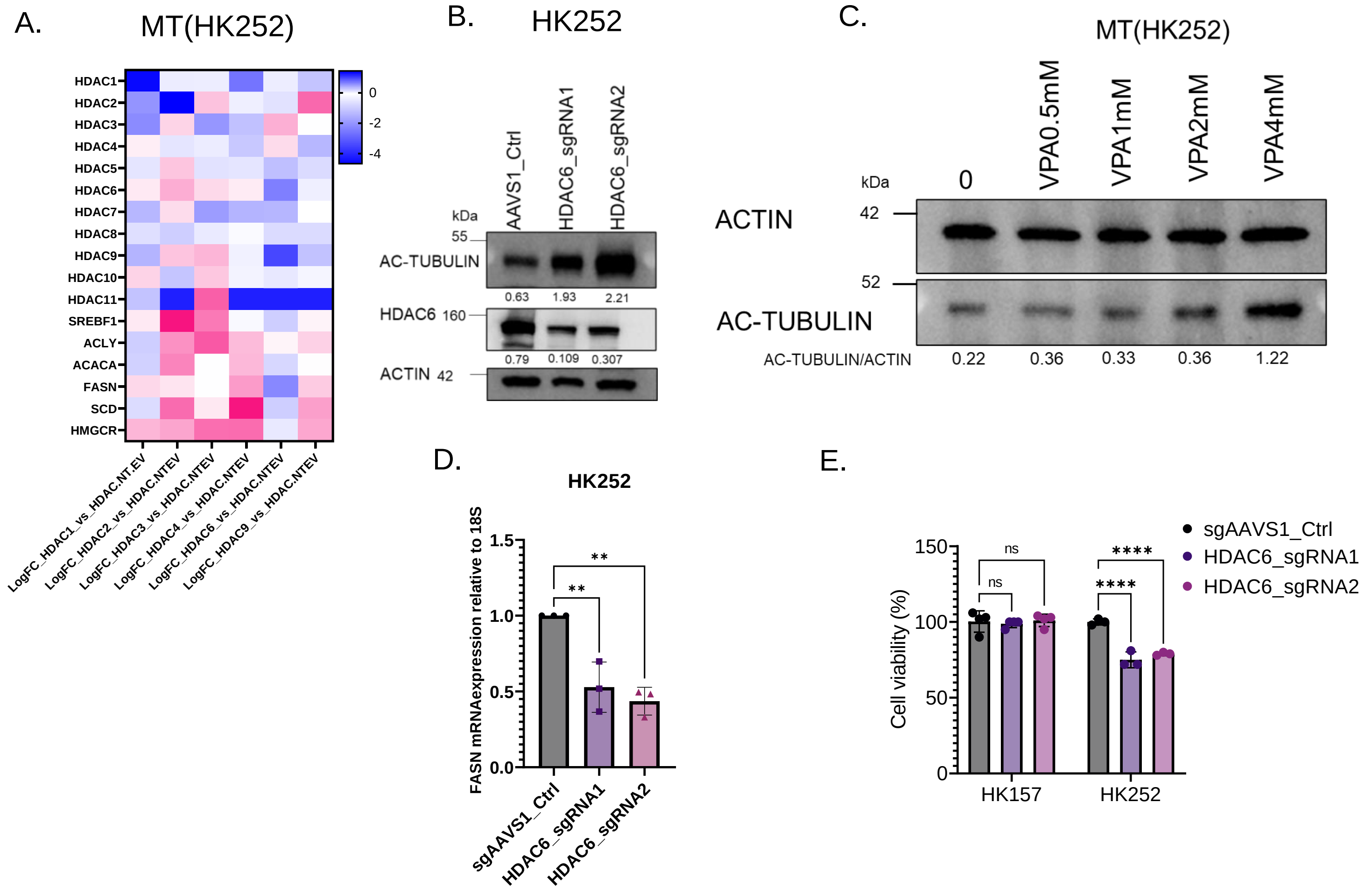

A.
MT(HK252)
HDAC1
HDAC2
HDAC3
HDAC4
HDAC5
HDAC6
HDAC7
HDAC8
HDAC9
HDAC10
HDAC11
SREBF1
ACLY
ACACA
FASN
SCD
HMGCR
0
-2
-4
LogFC_HDAC1_vs_HDAC.NT.EV
LogFC_HDAC2_vs_HDAC.NTEV
LogFC_HDAC3_vs_HDAC.NTEV
LogFC_HDAC4_vs_HDAC.NTEV
LogFC_HDAC6_vs_HDAC.NTEV
LogFC_HDAC9_vs_HDAC.NTEV
B.
HK252
AAVS1_Ctrl
HDAC6_sgRNA1
HDAC6_sgRNA2
kDa
55
AC-TUBULIN
0.63
1.93
2.21
HDAC6
160
0.79
0.109
0.307
ACTIN
42
C.
MT(HK252)
kDa
0
VPA0.5mM
VPA1mM
VPA2mM
VPA4mM
ACTIN
42
52
AC-TUBULIN
AC-TUBULIN/ACTIN
0.22
0.36
0.33
0.36
1.22
D.
HK252
FASN mRNAexpression relative to 18S
1.5
1.0
0.5
0.0
**
**
sgAAVS1_Ctrl
HDAC6_sgRNA1
HDAC6_sgRNA2
E.
sgAAVS1_Ctrl
HDAC6_sgRNA1
HDAC6_sgRNA2
Cell viability (%)
150
100
50
0
ns
ns
****
****
HK157
HK252

SFig7: HDACs are involved in regulation of lipogenic genes.

A. Heatmap showing expression of lipogenic genes after HDAC knockdown in IDH MT cell line HK252

B. Representative WB showing tubulin acetylation and HDAC6 protein expression after HDAC6 knockdown with two CRISPR sgRNA

C. Representative WB showing acetylation of tubulin after treatment with VPA for 4 days

D. FASN mRNA expression after HDAC6 knockdown

E. Relative cell viability of HK252 and HK157 after HDAC6 knockdown. 1-way ANOVA, **P value< 0.01, ****P value<0.0001; Results are expressed as the mean ±Standard deviation with individual datapoints illustrated.

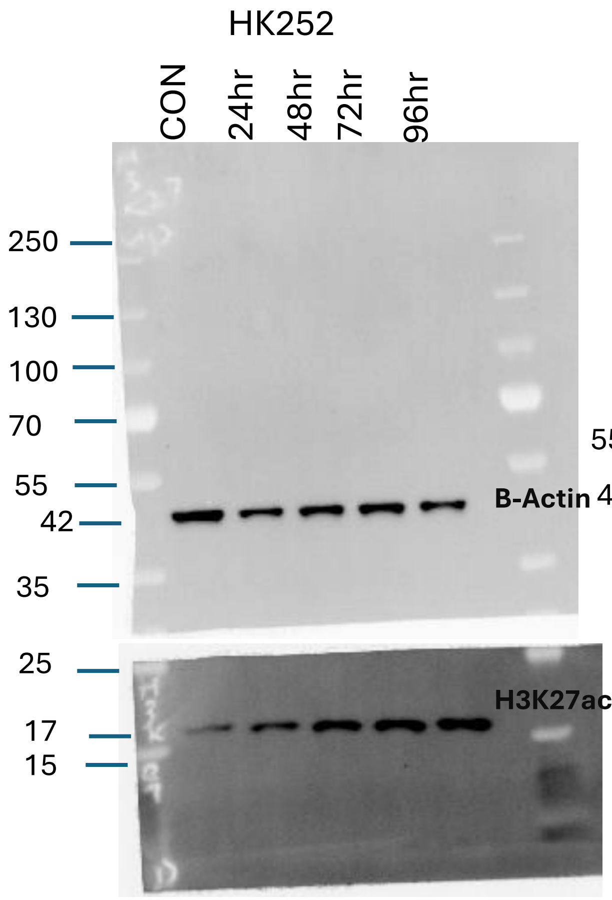

Fig1B

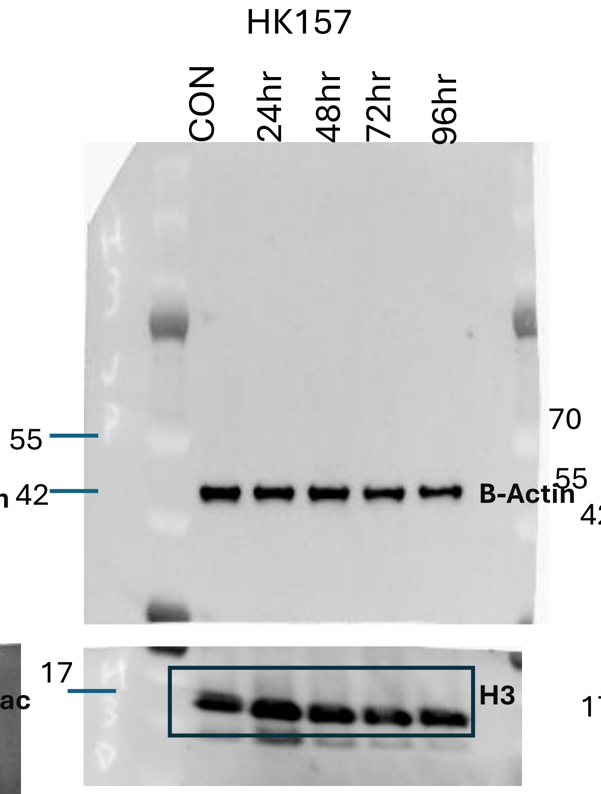

Fig1A

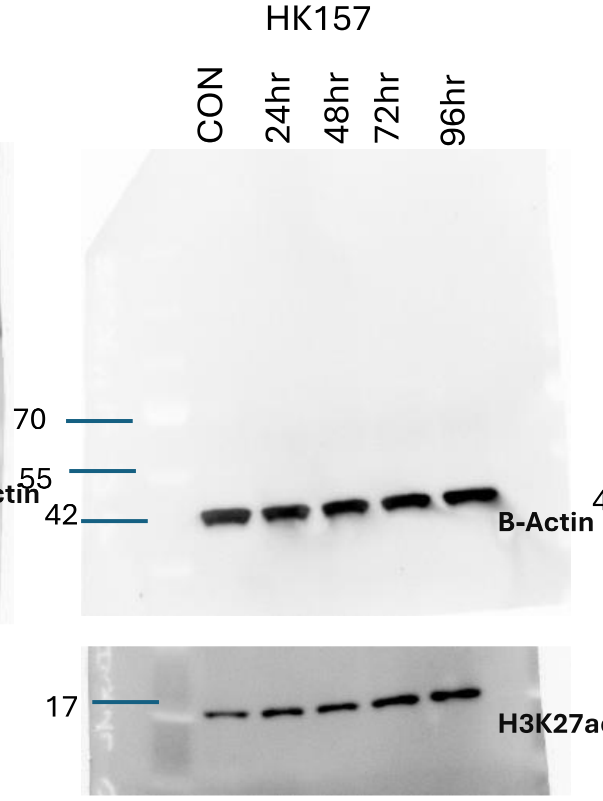

Fig1A

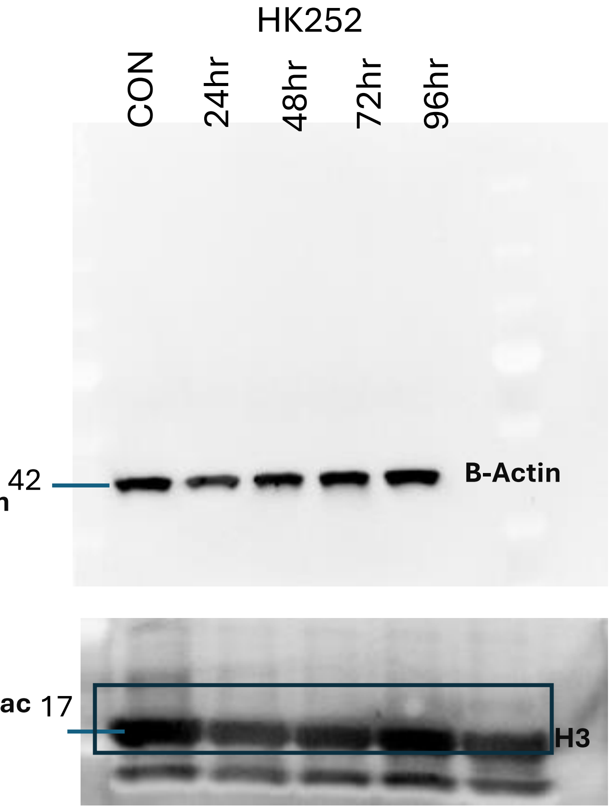

Fig1B

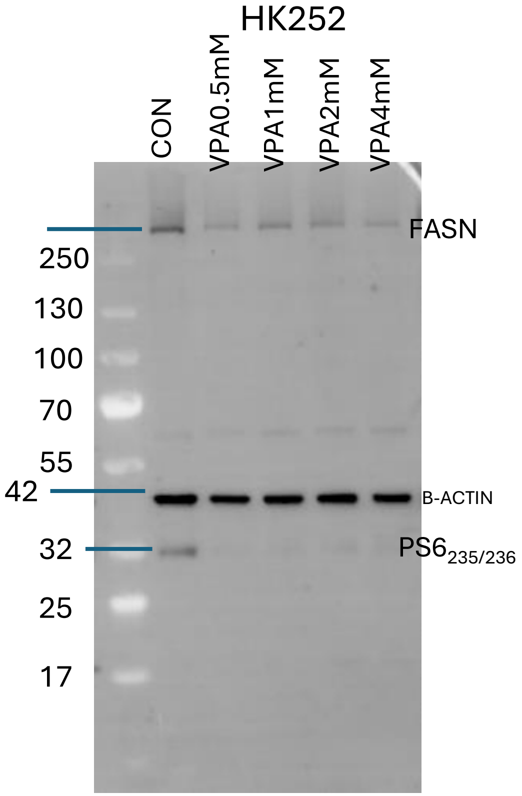

Fig4B

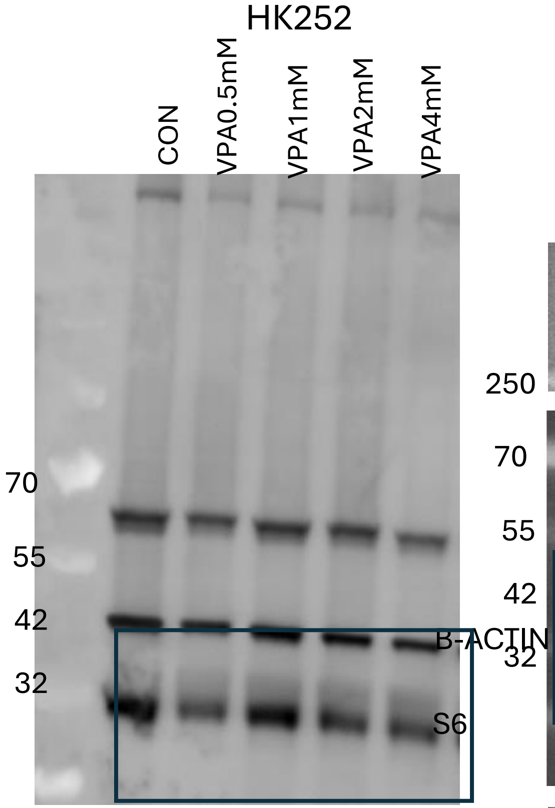

Fig4B

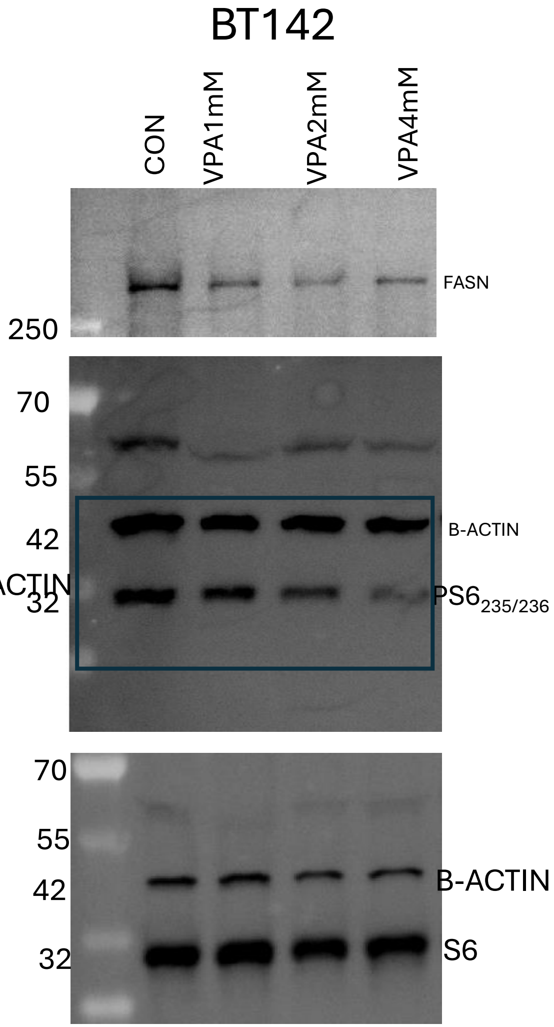

Fig4C

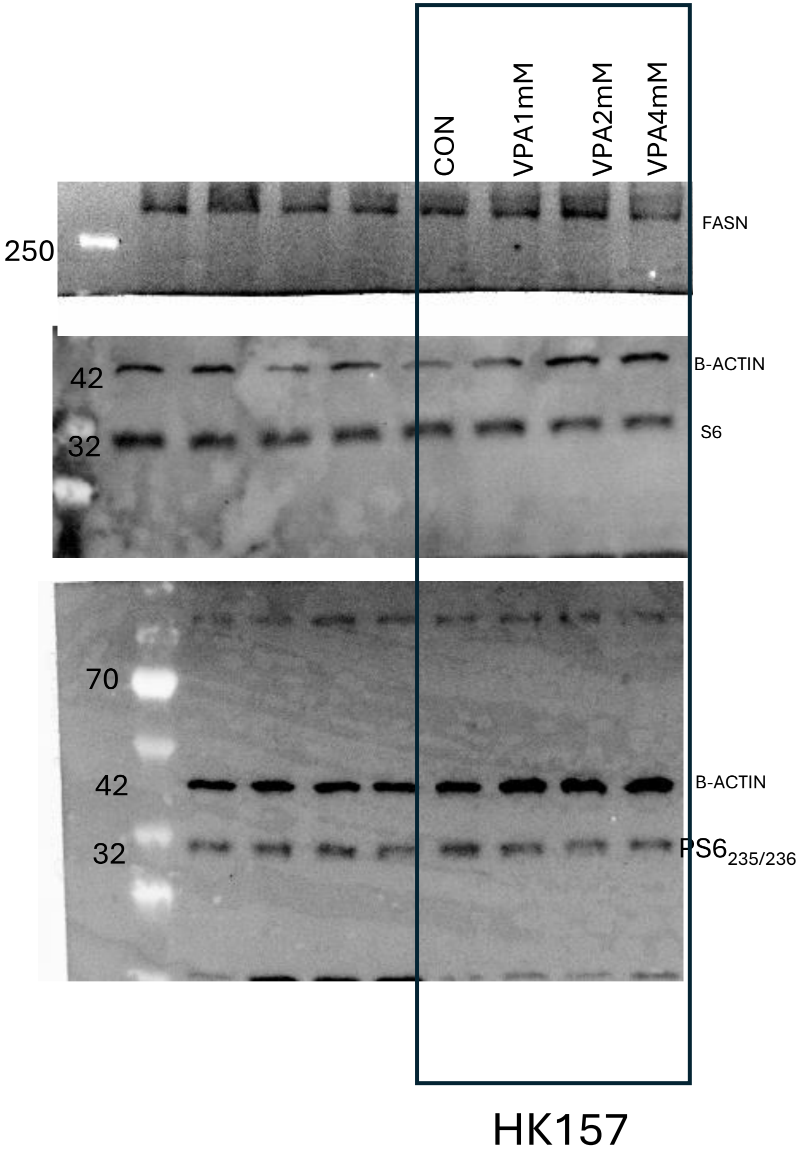

Fig4A

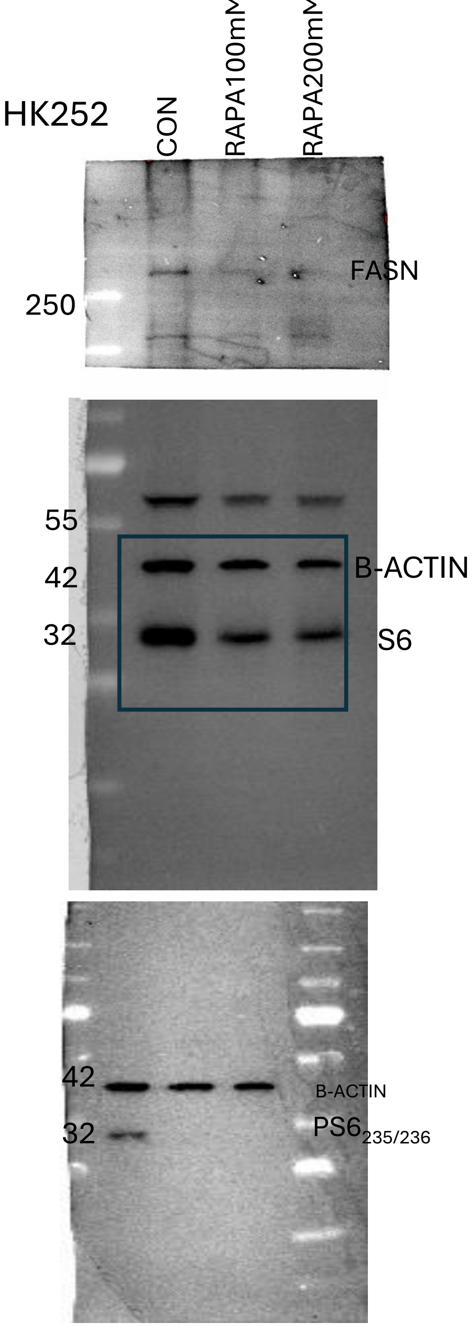

Fig4E

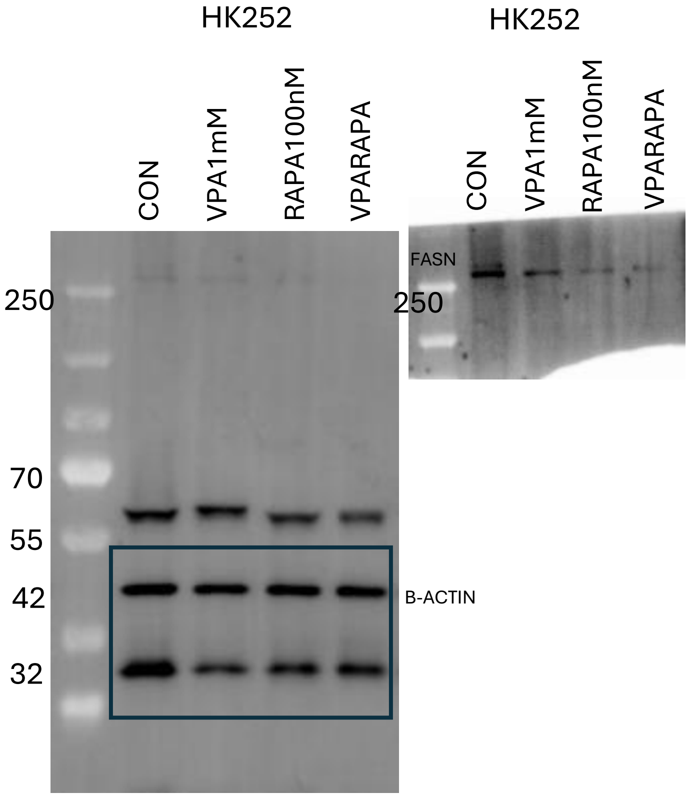

Fig4F

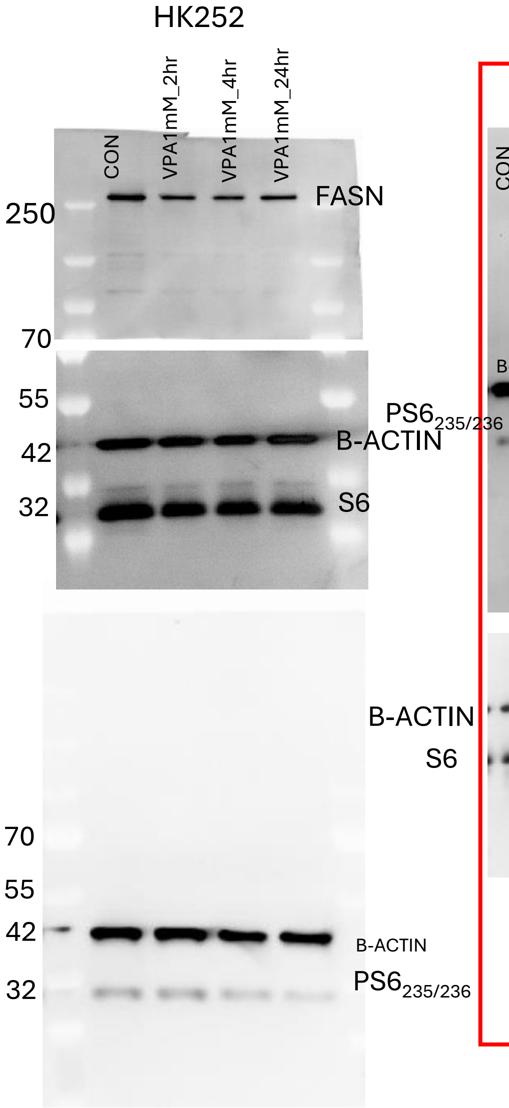

Fig4D

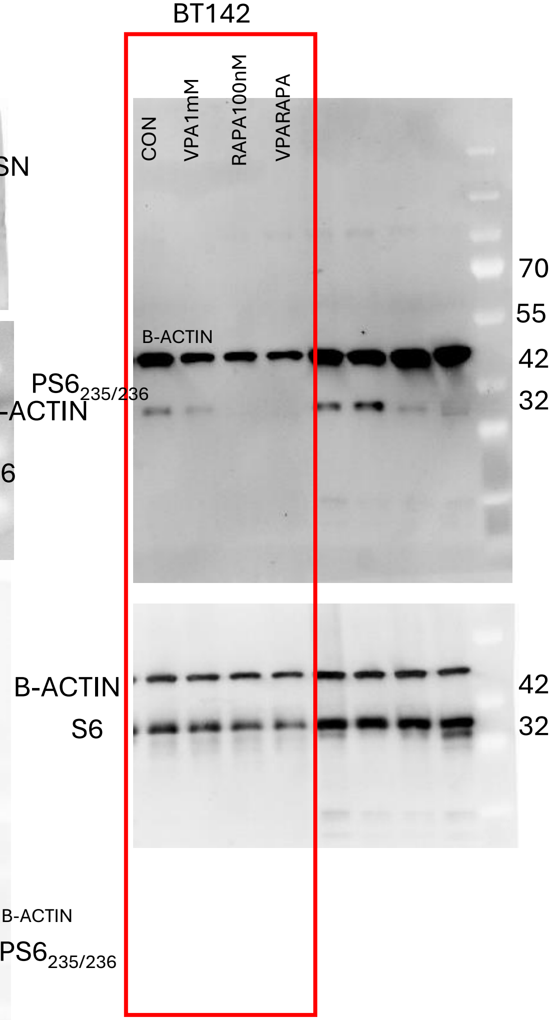

Fig4H

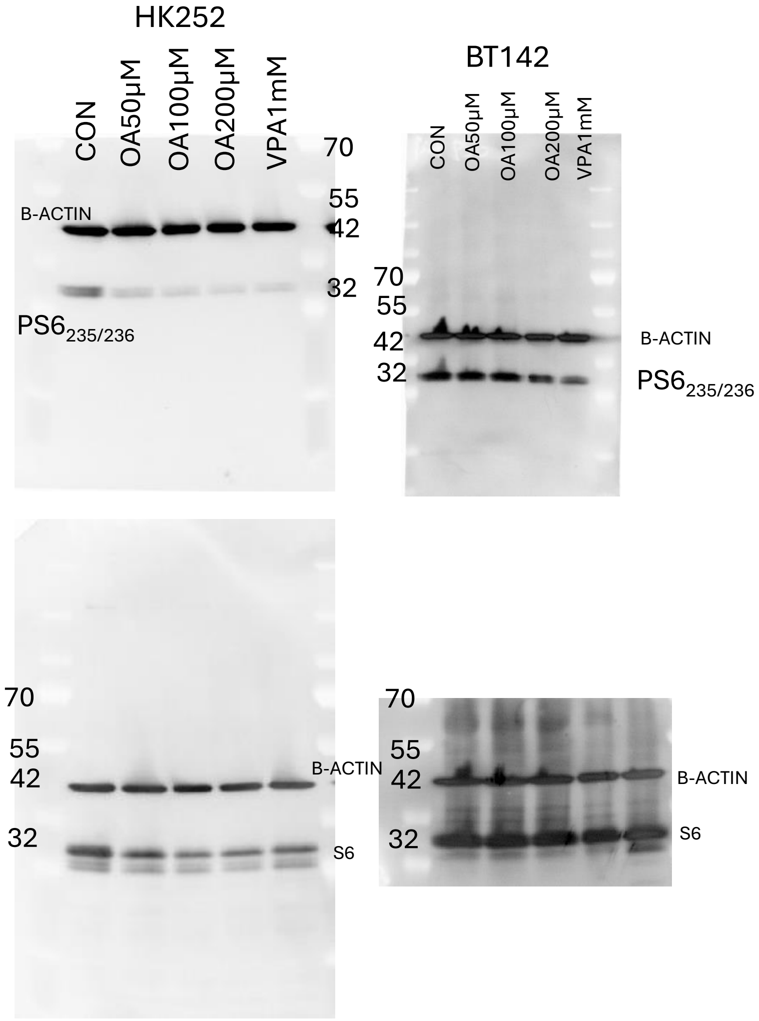
HK252
CON
OA50μM
OA100μM
OA200μM
VPA1mM
70
55
42
32
B-ACTIN
PS6235/236
BT142
CON
OA50μM
OA100μM
OA200μM
VPA1mM
70
55
42
32
B-ACTIN
PS6235/236
70
55
42
32
B-ACTIN
S6
70
55
42
32
B-ACTIN
S6

Fig7O

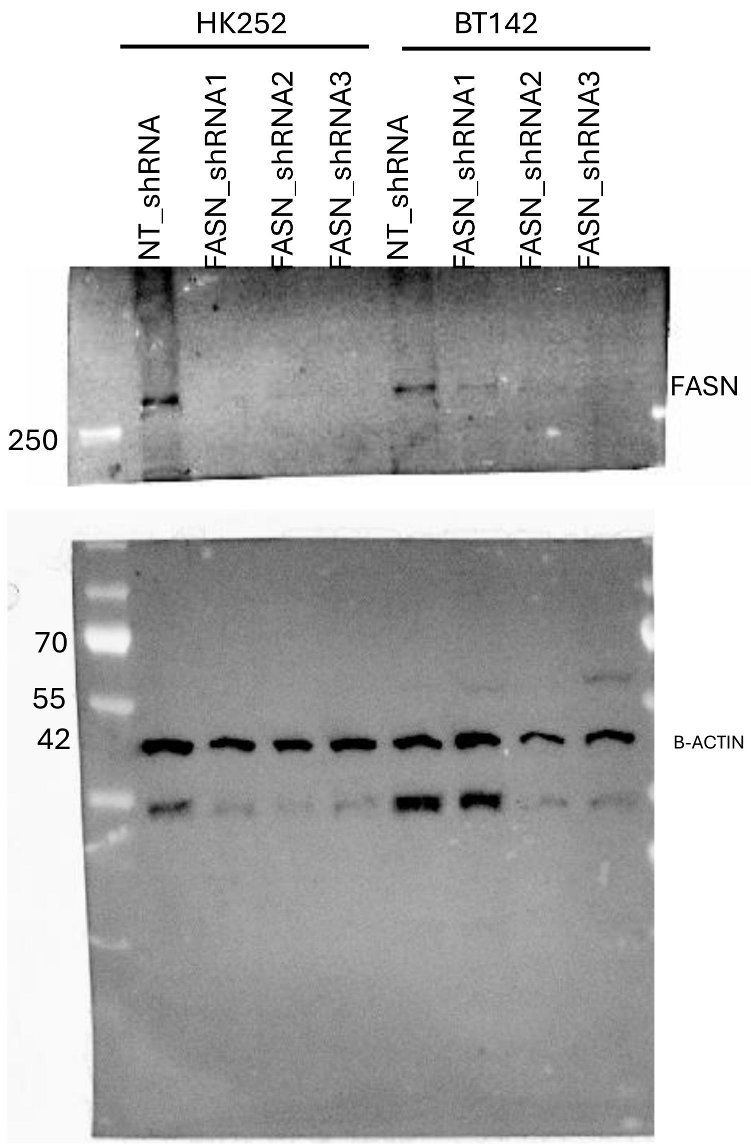
HK252
BT142
NT_shRNA
FASN_shRNA1
FASN_shRNA2
FASN_shRNA3
NT_shRNA
FASN_shRNA1
FASN_shRNA2
FASN_shRNA3
FASN
250
70
55
42
B-ACTIN

Fig8A

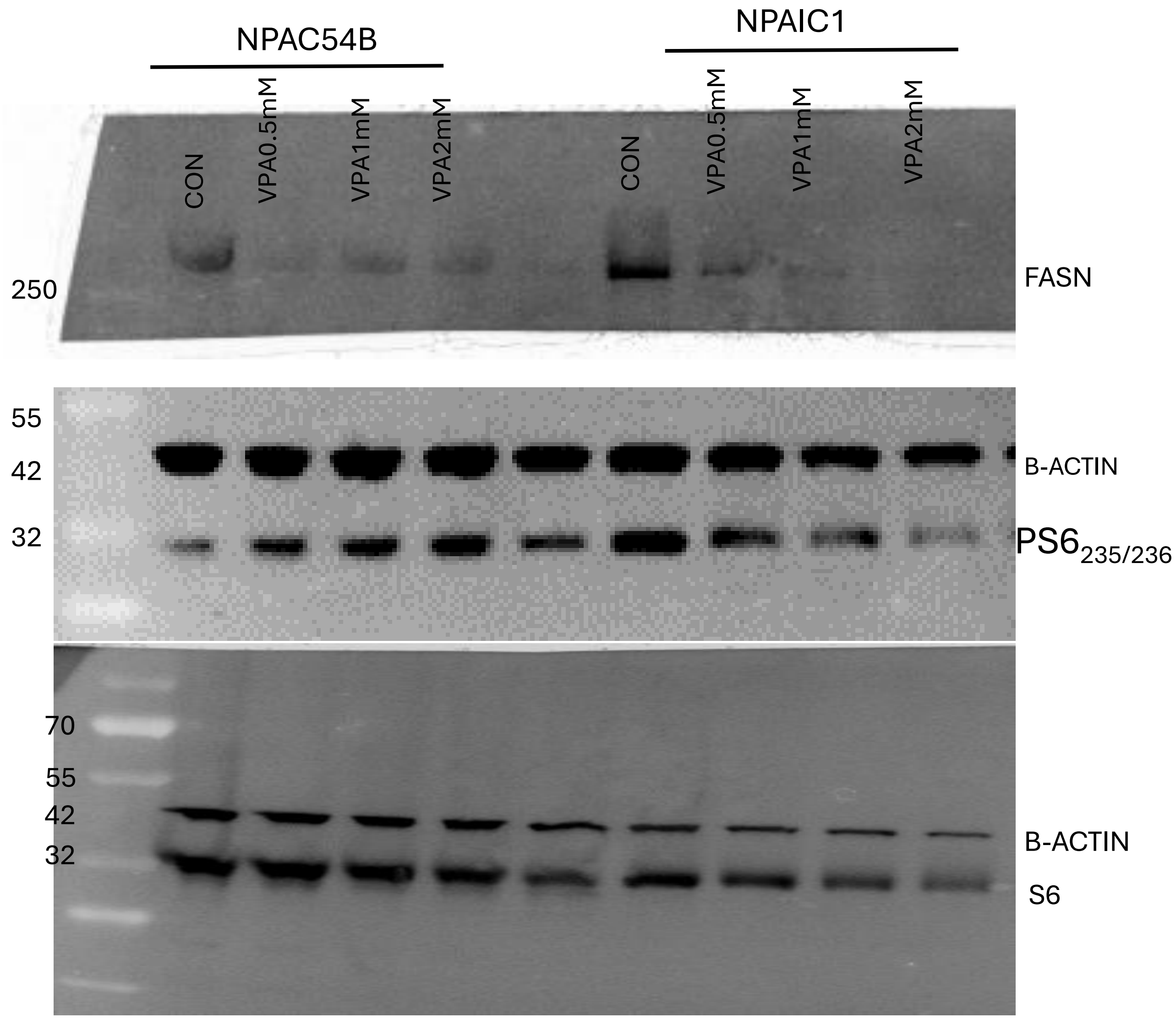
NPAC54B
NPAIC1
CON
VPA0.5mM
VPA1mM
VPA2mM
CON
VPA0.5mM
VPA1mM
VPA2mM
250
FASN
55
42
32
B-ACTIN
PS6 235/236
70
55
42
32
B-ACTIN
S6

SFig3

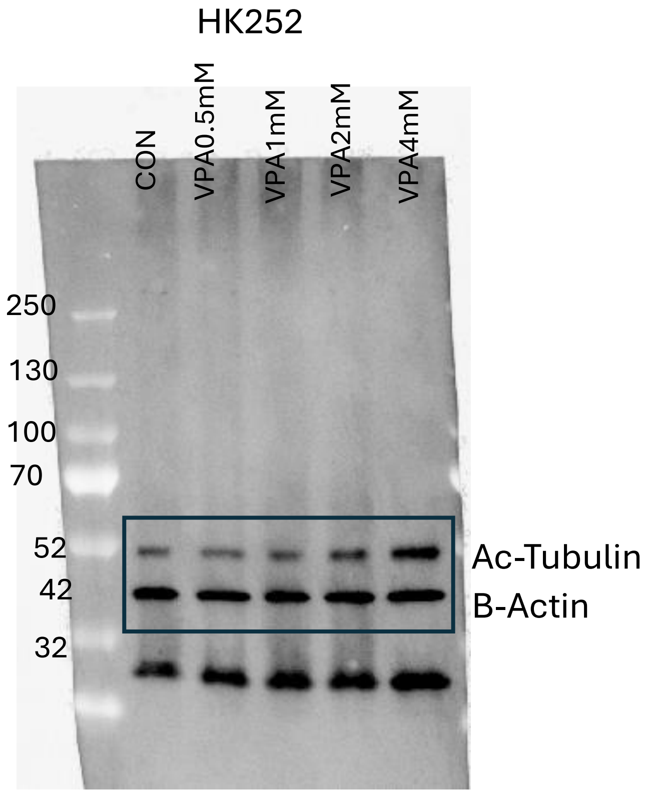

SFig7C

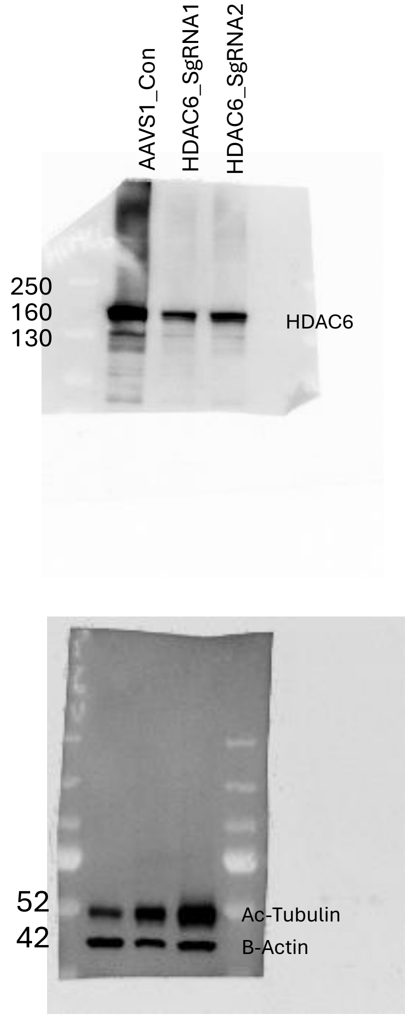

SFig7B
